# Metformin: The Answer to Cancer in a Flower? Current Knowledge and Future Prospects of Metformin as an Anti-Cancer Agent in Breast Cancer

**DOI:** 10.3390/biom9120846

**Published:** 2019-12-09

**Authors:** Samson Mathews Samuel, Elizabeth Varghese, Peter Kubatka, Chris R. Triggle, Dietrich Büsselberg

**Affiliations:** 1 Department of Physiology and Biophysics, Weill Cornell Medicine-Qatar, Education City, Qatar Foundation, Doha 24144, Qatar; elv2007@qatar-med.cornell.edu; 2 Department of Medical Biology, Jessenius Faculty of Medicine, Comenius University in Bratislava, 03601 Martin, Slovakia; peter.kubatka@uniba.sk; 3 Department of Pharmacology, Weill Cornell Medicine-Qatar, Education City, Qatar Foundation, Doha 24144, Qatar; cht2011@qatar-med.cornell.edu

**Keywords:** anti-cancer therapy, cancer, combination therapy, metformin, natural compounds, resistance

## Abstract

Interest has grown in studying the possible use of well-known anti-diabetic drugs as anti-cancer agents individually or in combination with, frequently used, chemotherapeutic agents and/or radiation, owing to the fact that diabetes heightens the risk, incidence, and rapid progression of cancers, including breast cancer, in an individual. In this regard, metformin (1, 1-dimethylbiguanide), well known as ‘Glucophage’ among diabetics, was reported to be cancer preventive while also being a potent anti-proliferative and anti-cancer agent. While meta-analysis studies reported a lower risk and incidence of breast cancer among diabetic individuals on a metformin treatment regimen, several in vitro, pre-clinical, and clinical studies reported the efficacy of using metformin individually as an anti-cancer/anti-tumor agent or in combination with chemotherapeutic drugs or radiation in the treatment of different forms of breast cancer. However, unanswered questions remain with regards to areas such as cancer treatment specific therapeutic dosing of metformin, specificity to cancer cells at high concentrations, resistance to metformin therapy, efficacy of combinatory therapeutic approaches, post-therapeutic relapse of the disease, and efficacy in cancer prevention in non-diabetic individuals. In the current article, we discuss the biology of metformin and its molecular mechanism of action, the existing cellular, pre-clinical, and clinical studies that have tested the anti-tumor potential of metformin as a potential anti-cancer/anti-tumor agent in breast cancer therapy, and outline the future prospects and directions for a better understanding and re-purposing of metformin as an anti-cancer drug in the treatment of breast cancer.

## 1. Background and Introduction

The reinforced link between diabetes and cancer and/or breast cancer has generated interest in studying the effects of widely prescribed anti-hyperglycemic/anti-diabetic drugs on the risk, incidence, progression, response to therapy, resistance, and post-therapeutic relapse in cancers. The incidence and rising rates of diabetes is a serious concern in the medical field around the globe. Worldwide, the World Health Organization (WHO) data estimate nearly 422 million diabetes affected individuals in 2014, a sharp rise when compared to the 108 million diabetes affected individuals in 1980 [1]. Data projections suggest that the global diabetes prevalence of 8.8% in 2017 will further increase to 9.9% by 2045 [2]. While it is well established that diabetes is linked to a higher risk of cardiovascular diseases, hepatic and renal complications, and nerve damage, much less appreciated is the fact that diabetes can be linked to a higher risk, incidence, progression, and post-treatment prognosis of different cancers [3,4,5,6,7]. More recently, owing to the many common risk factors attributable to both diabetes and cancer a convincing link was established, by several epidemiological studies, between the occurrence of diabetes and the higher risk and incidence of many different cancers, including the various types of breast cancer [5,8,9,10,11,12]. In this regard, particularly in breast cancers, while insulin and insulin analogues used to treat diabetes propagated tumor growth through the induction of angiogenesis and activation of mitogenic signaling mechanisms and drugs such as thiazolidinediones do not appear to have a significant anti-cancer effect, metformin on the other hand exhibited significant anti-proliferative and anti-cancer effects [5].

Metformin (1, 1-dimethylbiguanide) has its history traced back to the 18th century (year 1772), when *Galega officinalis* (commonly known as French Lilac/Goat’s Rue/Spanish Safonin/False Indigo) was used to treat symptoms which was later attributed to diabetes [13,14]. While the hypoglycemic activity of *Galega officinalis* was attributed to the guanidine component by the 1800s, the apparent toxicity associated with the clinical use of guanidine led to synthesis, testing, and use of several biguanides, including dimethylbiguanide, for their glucose-lowering and anti-malarial effects and for the treatment of influenza in the late 1920s [13,14]. It was then in 1957 that Dr. Jean Sterne published his studies on metformin and proposed its clinical development and the name ‘Glucophage’ (meaning glucose-eater) for metformin [13,14]. Metformin was thrust into the limelight as a better anti-hyperglycemic drug by the late 1970s, when its cousins, the biguanides such as phenformin and buformin (which had more potent glucose-lowering effect), were associated with lactic acidosis and had to be discontinued in medicinal practice [13,14]. Metformin on the other hand reportedly has only mild to moderate side effects such as nausea, vomiting, and diarrhea, which can be rectified by treatment dosage adjustments [15]. However, predominantly in elderly individuals, with heart failure, hypoxia, sepsis, renal and hepatic comorbidities, and dehydration, metformin administration can lead to lactic acidosis in rare cases [15,16,17,18]. The confirmed anti-hyperglycemic effect (without causing hypoglycemia) and the favorable safety prolife when compared to phenformin and buformin helped metformin claim the title as the ‘most widely prescribed and first-line oral anti-diabetic drug’ and manages to keep that title 62 years after its first clinical use in the treatment and management of type 2 diabetes [13,14,19].

**Figure 1:**
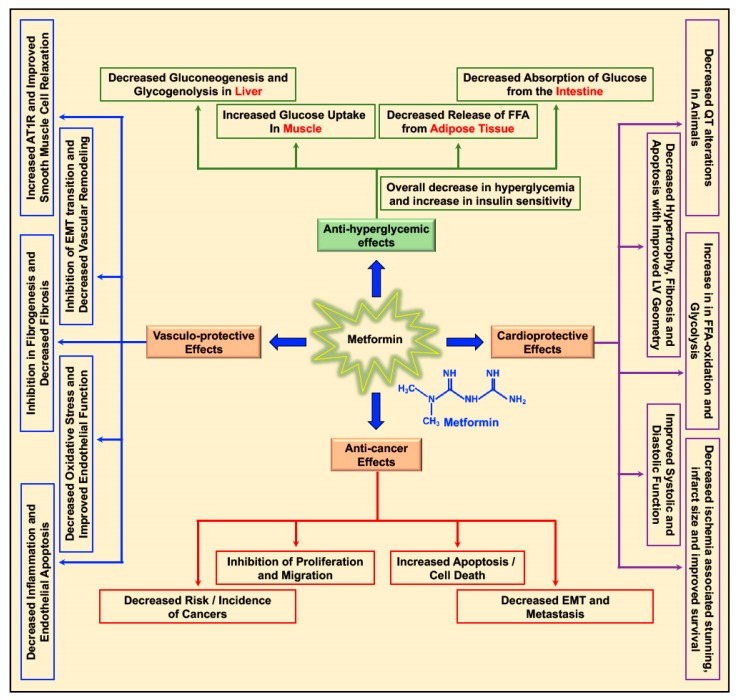
Multifaceted benefits of metformin: Metformin reduces blood glucose levels in circulation by decreasing gluconeogenesis and glycogenolysis in the liver, decreasing the intestinal absorption of glucose, reducing the release of free fatty acids (FFA) from adipose tissue, and increasing glucose utilization by the muscle. Metformin exerts its cardioprotective effects by increasing cardiac FFA oxidation and glycolysis, reducing ischemia-associated stunning and infarct size, decreasing cardiac hypertrophy, apoptosis, and fibrosis, thereby improving cardiac functions (systolic and diastolic). Metformin’s vasculo-protective effect is accounted for by its effect on reducing inflammation, endothelial apoptosis, oxidative stress, and fibrosis of the vasculature, improving both endothelial and smooth muscle cell function and inhibiting epithelial mesenchymal transition (EMT) transition, thus curbing vascular remodeling and causing overall improvement of vascular function. In addition, metformin exerts its anti-cancer effects by decreasing incidence of different cancers and inhibition of proliferation and migration of cancer cells, activation of apoptosis, and reducing EMT and metastasis.

Metformin decreases the levels of blood glucose by decreasing gluconeogenesis and glycogenolysis in the liver, decreasing the intestinal absorption of glucose, reducing the release of free fatty acids (FFA) from adipose tissue, and increasing glucose utilization by the muscle (Figure 1) [20]. Apart from its glucose-lowering effect, metformin was studied for its cardioprotective and vasculo-protective effects and more recently for its effects as a cancer preventive and anti-cancer/anti-tumor agent in different cancers (Figure 1) [5,20,21]. Depending on patient prolife and various disease conditions or stages, metformin treatment-associated beneficial effects in the treatment of hepatic diseases [22,23,24,25], renal damage and disorders [26], neurodegenerative diseases [27,28,29], and bone disorders [30] were reported. In addition, metformin treatment-related antiaging effects, delay in the onset of age-related disorders, and improvement in longevity (lifespan) were reported in *C. elegans*, insects, and rodents [31,32,33,34].

Interest has grown in studying the possible use of metformin as an anti-cancer/anti-tumor agent individually or in combination with frequently used chemotherapeutic agents and/or radiation. Epidemiological studies and meta-analysis data suggest that diabetic individuals on a metformin treatment regimen, to control their blood glucose levels, have a lower risk of developing cancers of all types and additionally individuals who are both diabetic and suffering from cancer and on metformin treatment have an improved response to chemotherapy and radiation therapy, better prognosis, and higher survival rates when compared to those who do not take metformin [5,35,36,37,38,39,40,41,42]. In cancer cells, the ability of metformin to alter cancer metabolism and mitochondrial function and to modulate intracellular signaling activity related to key oncogenic pathways such as the Ras/Raf/MEK/ERK, PI3K/Akt, and mTOR pathways, retards cancer cell growth, proliferation, migration, increases cell death, and inhibits EMT, invasion, and metastasis [36,43,44,45,46,47]. While the activation of AMPK seems to be key to the many of the beneficial anti-cancer effects of metformin, AMPK independent effects have also been reported [5,36,43,44,45,47]. However, most of the mechanistic data on the anti-cancer effects of metformin were derived from in vitro experiments using cancer cell lines and thus may not reflect the mode of action of metformin in an in vivo or clinical setting.

Several epidemiological and meta-analysis data and in vitro, pre-clinical, and clinical studies also link the beneficial outcomes in the treatment of different forms of breast cancer to metformin treatment either individually or in combination with chemotherapy and radiation therapy. In the current article, we discuss the biology of metformin and its molecular mechanism of action, the existing cellular, pre-clinical, and clinical studies that have tested the anti-tumor potential of metformin as a potential anti-cancer/anti-tumor agent in breast cancer therapy and outline the future prospects and directions for a better understanding and re-purposing of metformin as an anti-cancer drug in the treatment of breast cancer. We aim to encourage the scientists working with metformin in breast cancer to address unanswered questions pertaining to areas such as breast cancer specific therapeutic dosing of metformin, specificity of metformin to breast cancer cells at high concentrations, resistance of specific forms of breast cancer to metformin therapy, the efficacy of combinatory therapeutic approaches, post-therapeutic relapse of the disease, and efficacy in breast cancer prevention in non-diabetic individuals.

## 2. Biology of Metformin and Molecular Mechanism of Action

In a type 2 diabetic individual who receives metformin orally, the concentration of metformin in the hepatic circulation may reach 50 μM; with the peak plasma concentration of metformin at 20 μM [5,48,49]. The hydrophilic and cationic nature of metformin at physiological pH makes it highly unlikely that metformin rapidly diffuses through the cell membrane and exerts it effect on cell function. In addition, the kidneys carry out the elimination of unaltered metformin through the urine [50]. Hence, it is evident that metformin requires the presence and support of transporter molecules for its absorption, distribution, and elimination to exert its biological function. In this regard, the organic cation transporters 1, 2, and 3 (OCT1, OCT2, and OCT3), the plasma membrane monoamine transporter (PMAT), and multidrug and toxin extrusion protein 1 and 2 (MATE1 and MATE2) transporters are reported to play key roles in transporting metformin into and out of the cell in the intestine, liver, and kidney [50,51,52,53,54,55,56,57]. The thiamine transporter 2 (THTR2) also plays a role in intestinal absorption and renal re-absorption of metformin [58]. Alterations in the OCT1 gene reduced hepatic uptake of metformin and reduced the efficacy of metformin in reducing blood glucose levels by the inhibiting gluconeogenesis and glycogenolysis [59,60].

**Figure 2:**
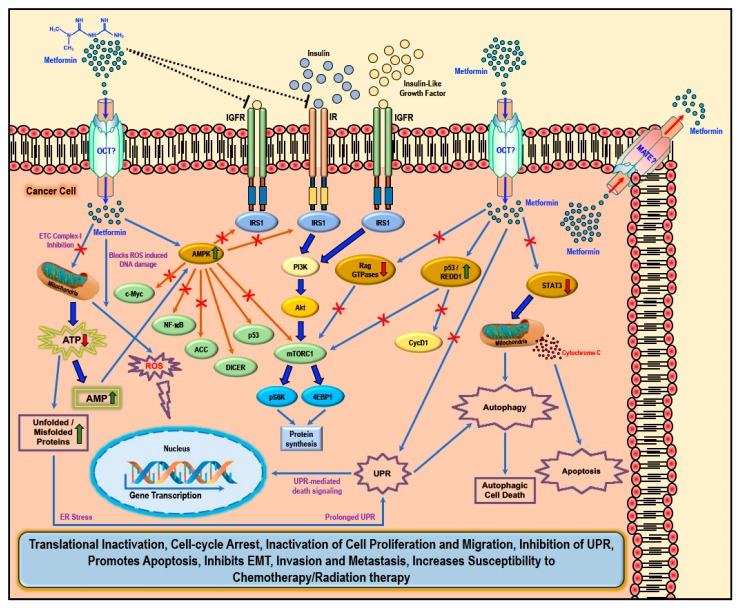
Cellular anti-cancer/anti-tumor effects of metformin: The hydrophilic and cationic metformin is transported into the cell via the organic cation transporters (OCT), which support the intracellular accumulation of metformin. The anti-proliferative activity of metformin in several cancers is at least in part attributed to its ability to reduce the levels of insulin/IGF1, which in turn inhibits the insulin/IGF1 mediated molecular pathways that support tumor initiation and progression. Metformin treatment directly activates AMPK and the ‘AMPK dependent’ effects include inhibition of c-Myc, NF-κB, and mammalian target of rapamycin-C1 (mTORC1) pathways and acetyl Co-A carboxylase (ACC)-dependent lipogenesis pathways while activating the p53 pathway and DICER-mediated miRNA synthesis. Metformin, albeit at high concentrations, is also known to inhibit the mitochondrial Complex 1 of the electron transport chain (ETC) thereby reducing ATP, levels which increases the AMP/ATP ratio further leading to AMPK activation. A decrease in ATP/energy levels can also lead to mismanaged protein folding mechanisms leading to the accumulation of unfolded or misfolded proteins and prolonged unfolded protein response (UPR) without rectification of endoplasmic stress triggers apoptosis through multiple mechanisms, which include activation of UPR mediated apoptotic/death signaling and activation of autophagy and subsequent autophagic cell death. AMPK independent metformin treatment-associated anti-cancer effects are mediated by Rag GTPases, REDD1, and STAT3. Overall metformin treatment in cancer cells causes translational inactivation, cell-cycle arrest, inactivation of cell proliferation and migration, inhibition of UPR, promotes apoptosis, inhibits EMT, invasion, and metastasis, and increases susceptibility to chemotherapy/radiation therapy.

While several studies have reported various ‘AMPK dependent’ and ‘AMPK independent’ mechanisms for the anti-cancer/anti-tumor effects of metformin in cancer therapy, these anti-cancer effects of metformin were only observed at very high concentrations (>5 mM) and fall short of explaining how such high concentrations enters the cancer cells and exerts its anti-neoplastic effect. Studies have implicated that the susceptibility and/or resistance of cancer cells to metformin treatment is dependent on the varying levels of the cell surface metformin transporters. Overexpression of OCTs that contribute to intracellular accumulation of metformin in cancers would make them susceptible to metformin treatment (which should explain the high concentrations of metformin required for anti-cancer treatment) while the overexpression of MATE transporters that contribute to the extrusion of metformin out of the cell would render the cancer cell resistant to metformin treatment (Figure 2) [61,62,63,64].

There are two general mechanisms that could explain the putative anti-cancer effects of metformin. The ‘indirect’ anti-cancer effects of metformin arise from its ability to reduce insulin resistance, insulin levels, and fasting glucose levels [65]. Physiologically, insulin and insulin-like growth factor-1 (IGF1) largely regulate carbohydrate and lipid metabolism and storage and protein synthesis via transmembrane receptor binding and activation of receptor tyrosine kinase and subsequent activation of intracellular insulin receptor substrate-1 (IRS1); however, insulin and IGF1-mediated signaling pathways are also implicated in pathogenesis and progression of several cancers via the activation of the Ras/Raf/MEK/ERK, PI3K/Akt/mTORC1, and GSK3β/β-catenin pathways [5,44]. Metformin reduces blood glucose levels in circulation by decreasing gluconeogenesis and glycogenolysis in the liver, decreasing the intestinal absorption of glucose, reducing the release of FFA from adipose tissue, and increasing glucose utilization by the muscle. Lower blood glucose concentration in turn decreases the synthesis and secretion of insulin by the β-cells of the pancreas and reduces the levels of insulin in circulation. The anti-proliferative activity of metformin in several cancers is at least in part attributed to its ability to reduce the levels of insulin/IGF1, which in turn inhibits the insulin/IGF1 mediated molecular pathways that support tumor initiation and progression rather than a direct anti-proliferative/anti-cancer action (Figure 2) [44].

Metformin also exhibited ‘direct’ anti-cancer effects in many different cancer related studies. Since cancer cells are known to utilize glucose rapidly through glycolysis (Warburg effect) to meet their energy needs when compared to normal cells, metformin-mediated decrease in glucose levels should also curb tumor growth, although reports suggest that cancer cells use alternative sources of energy when starved of glucose or when glycolysis is inhibited [45,66,67,68]. Furthermore, AMPK inhibits IRS1 mediated IR and IGFR oncogenic signaling via PI3K/Akt/mTOR, which, potentially, also contributes to the anti-cancer effect of metformin (Figure 2) [69,70,71].

Several experimental studies reported the ‘direct’ anti-cancer effects of metformin, which are distinct from its ‘indirect’ anti-cancer effects that are related to its anti-hyperglycemic actions, inhibition of hepatic gluconeogenesis, and reduction of insulin signaling [72,73,74]. In cancer cells, aberrant signaling mechanisms were reported involving key proteins and their related pathways associated with protein synthesis and survival (mTOR, c-Myc, and NF-κB), lipid synthesis (ACC), DNA damage repair and apoptosis (p53), and miRNA synthesis and function (DICER). Each of these proteins and their modulation/regulation can impact the incidence, growth, and progression of malignant tumors. Mammalian target of rapamycin-C1 (mTORC1) is activated and upregulated by nutrients, growth factors and energy and stress signals, and key signaling pathways (PI3K, MAPK, and AMPK) and is inhibited by rapamycin [75,76,77]. Activation of mTORC1 regulates cellular protein synthesis and cell survival through the phosphorylation of its substrates, 4EBP1, and p70S6K [75,76,77]. The central role of mTOR in regulating cellular protein synthesis and cell survival explains the association of an overactive mTOR pathway cancer [75,76,77]. Several mTOR inhibitors were successfully tested for the treatment of various cancers [75,76,77]. Cellular Myc (c-Myc) is a well-studied oncogene which was constitutively overexpressed in various cancers [78,79]. Myc reportedly regulates an array of cellular functions that support cancer cell growth and progression including transcription of several oncogenes, translation, cell cycle progression, cell proliferation, differentiation and survival, ribosome biogenesis, signal transduction, and also cancer stem cell-related signaling and resistance to cancer treatment [78,79,80]. NF-κB activation, similarly, promotes cancer growth and by regulating transcription of genes that support cell proliferation and survival, angiogenesis, tumor progression, and metastasis [81,82,83]. The DNA repair and pro-apoptotic nuclear transcription factor, p53, is vital to tumor suppression [84]. Loss of p53 function and mutations in the p53 gene are notably the cause for the incidence and progression of many different cancers by supporting cell proliferation and survival, metabolism, genome instability, pro-survival autophagy, and metastasis in addition to conferring therapeutic resistance to cancer cells [84,85,86]. The RNase enzyme, DICER, that is important in processing the formation of function micro-RNAs (miRNA) was frequently downregulated in human cancers and was linked to cancer progression and cancer metastasis [87,88,89].

Metformin-mediated activation of AMPK and subsequent modulation and regulation of intracellular proteins and their functions can explain several of the biological functions as well as its anti-cancer/anti-proliferative effects that was observed in most cancer cells (Figure 2) [44,45,90,91,92,93]. Activation of AMPK in cancer cells is associated with inhibition of the mTORC1, c-Myc, and NF-κB pathways and activation of DICER and the p53 pathway, all of which reportedly exert tumor suppressive, anti-proliferative, anti-migratory, and pro-apoptotic effects through various intracellular mediators, activation of anti-oncogenic genes, and downregulation of pro-oncogenic genes [94,95,96,97,98,99,100,101,102,103,104,105]. Metformin treatment-associated AMPK activation leads to the phosphorylation of tuberous sclerosis-2 (TSC2) or raptor and subsequent mTORC1 pathway inhibition, thereby reducing the cellular translational process/protein synthesis and overall cell survival [93,106,107,108,109,110]. AMPK also phosphorylates and inhibits acetyl CoA carboxylase (ACC), thereby reducing lipid biosynthesis (Figure 2) [111]. Inhibition of these anabolic processes of protein and lipid biosynthesis thus retards cancer cell growth and proliferation [112].

Furthermore, metformin can inhibit the mitochondrial respiratory chain complex 1 (Figure 2), thereby causing a reduction in the NADH oxidation and the proton gradient across the inner mitochondrial membrane subsequently reducing the rate of oxygen consumption [113,114]. Since cancer cells are highly glycolytic in nature (Warburg effect) and depend less on the oxidative phosphorylation for its energy needs (ATP), it can be argued that the effect of metformin as an inhibitor of the electron transport chain (ETC) complex 1 may be weak and reversible and may not impact the growth or proliferation of cancer cells [47]. However, any cancer cell that utilizes oxygen for mitochondrial respiration would produce mitochondrial ATP and, thus, a decrease in ATP production due to metformin-mediated inhibition of ETC complex 1 should be toxic to the cells [47]. Additionally, the increasing levels of AMP due to ETC complex 1 inhibition should also in turn activate AMPK (Figure 2) [46,115]. The decrease in ATP also contributes to the accumulation of unfolded/misfolded proteins [116,117,118]. The accumulation of misfolded or unfolded proteins turns on the endoplasmic reticulum stress/unfolded protein response (UPR) pathway [119]. Prolonged UPR and accumulation of unfolded proteins without rectification of endoplasmic reticulum stress triggers apoptosis through multiple mechanisms, which include activation of UPR-mediated apoptotic/death signaling and activation of autophagy and subsequent autophagic cell death [119]. Additionally, the ER stress mediated release of calcium (Ca^2+^) from the endoplasmic reticulum stores leads to Ca^2+^ accumulation in the mitochondria causing depolarization of permeability transition pore (PTP) and inducing apoptosis via the release of caspases [119].

Metformin treatment-associated ‘AMPK independent’ anti-cancer effects are mediated by regulated in DNA Damage-1 (REDD1; also known as DNA damage inducible transcript-4-DDIT4), Rag GTPases, and signal transducer and activator of transcription-3 (STAT3) (Figure 2). REDD1/DDIT4 is known to be inhibitor of mTOR signaling and thereby possess tumor suppressive properties by inhibition of protein synthesis and cell survival [120,121,122,123]. Metformin reportedly activated the p53/REDD1 axis to cause AMPK independent inhibition of mTOR in cancer cells (Figure 2) [124]. Activation of the p53/REDD1 pathway also reduces the expression of Cyclin D1, thereby reducing cell proliferation [125]. Rag GTPases, a sub-family of Ras-related GTPases, is involved in amino acid signaling mediated activation and functioning of the mTOR pathway [126,127,128]. Metformin treatment inhibits the mTOR pathway via the inhibition of Rag GTPases in cancer cells, which in turn reduces protein synthesis and causes cell cycle arrest (Figure 2) [125,129,130]. The aberrant activity of STAT3 has been implicated in promoting the pro-oncogenic functions such as initiation, progression, metastasis, and immune evasion in different cancers [131,132]. Overexpression of STAT3 contributes to cell survival, proliferation, cell cycle progression, anti-apoptosis, migration, invasion, angiogenesis, chemoresistance, immunosuppression, and self-renewal and differentiation of stem cells by regulating the expression of its downstream target genes [131,132]. Metformin treatment inhibited STAT3 nuclear translocation and exerted anti-proliferative, anti-metastatic, and pro-apoptotic effects in cholangiocarcinoma cells and breast cancer cells (Figure 2) [133,134].

The multifaceted ability of metformin to influence cancer cell growth and cancer progression through various molecular mechanisms, as discussed above, has made it an interesting candidate drug with potential in the treatment of breast cancer. In the following sections of the article, we briefly discuss the cellular, pre-clinical, and clinical studies that are currently testing metformin as a monotherapy or in combination with other chemotherapeutic drugs or phytochemicals/natural compounds for its efficacy as an anti-cancer/anti-tumor agent in the treatment of estrogen receptor (ER) positive, progesterone receptor (PR) positive, human epidermal growth factor receptor 2 (HER2) positive types of breast cancers, and triple negative breast cancers (TNBCs) [135].

## 3. Cellular and Pre-Clinical Data

The report by Evans JM, et al. in 2005 suggesting that metformin-treated diabetic patients had a reduced risk of cancers spurred a massive interest in studying the anti-cancer/anti-tumor effect of metformin giving rise to several in vitro and pre-clincal studies [39]. As elaborated in our article on the link between diabetes and breast cancer, insulin and IGFs promote the incidence and progression of breast cancer through several mechanisms that support translational activation, cell cycle progression, activation of cell proliferation and migration, inhibition of apoptosis, activation of EMT, increasing invasiveness and metastasis, and conferring resistance to chemotherapy [5]. The ability of metformin to promote glucose uptake by the muscles, increase insulin senitivity, reduce insulin levels, and thereby reduce blood glucose levels in itself reduces pre-neoplastic and neoplastic breast cell proliferation [136]. The fact that metformin has remained off patent since 2002, is easy to synthesize and economical, well-tolerated with very little side effects, and works at the level of the whole organism by reducing insulin levels and also directly on the tumor cells makes metformin an interesting drug of choice for the treatment of cancer [137].

Althought several of the studies reported metformin treatment-associated inhibition of cancer cell growth and proilferation, activation of cancer cell death, inhibition of invasion and metstasis, and tumor regression, these beneficial effects of metformin were observed at only significantly high concentrations (>5 mM), at least 100-fold higher than with the peak plasma concentration of metformin when administered orally for the treatment of type 2 diabetes [48,49]. As discussed earlier, the selective accumulation of metformin and therefore the sensitivity of cancer cells to metformin should depend on the levels of expression of the various transporters (OCTs, PMAT, and MATEs) in the cells [50,51,52,53,54,55,56,57,59,60]. In estrogen receptor (ER) and progesterone receptor (PR) positive MCF7 cells and triple negative breast cancer cells (BT20 and MDA-MB-468), overexpression of OCT3 was related to enhanced metformin uptake and anti-tumor efficacy, indicating that OCT3 supported the movement of metformin into the cancer cell [61]. In a rat model of 1-methyl-1-nitrosourea initiated mammary tumor, the intracellular accumulation of metformin, activation of AMPK, decrease in tumor volume and proliferation, and subsequent suppression of tumor progression correlated with the higher expression of OCT2, which also transported metformin into the cancer cell [62,64]. On the other hand, a study involving 19 different cancer cell lines identified that high MATE2 (which transports metformin out of the cell) expression levels correlated with the cancer cell resistance to the anti-proliferative effect of metformin [63].

The efficacy of metformin as an anti-cancer agent may also depend on the blood glucose levels in the treated individual. It was reported that in a normoglycemic condition, metformin treatment protected normal cells while causing cell cycle arrest in cancer cells of the breast [138]. Metformin treatment had little effect on breast cancer cells grown in hyperglycemic conditions, which promoted growth and aggressiveness of the cells [139]. On the other hand, metformin treatment in glucose-starved/deprived breast cancer cells induced cell death [139]. We have shown that the ability of metformin to inhibit the Akt/mTOR pathway, induce G_2_/M cell cycle arrest, and cell death in microvascular endothelial cells overexpressing VEGF, was enhanced under glucose-starved conditions [68,138]. This was shown to be true in other cancer cells as well [68,138]. In MCF7, SKBR3, and MDA-MB-231 cells, metformin effectively activated AMPK dependent apoptosis (partially independent of mTORC1) at physiological glucose conditions while the efficacy of metformin was lost under high glucose or amino acid rich conditions [140]. In fact, under nutrient poor conditions, metformin shifted the cellular glycolytic equilibrium through the AMPK dependent downregulation of pyruvate kinase M2 [140]. Under high glucose conditions, the energy derived from aerobic glycolysis promoted cell proliferation, conferred resistance to metformin treatment, and protected the triple negative breast cancer cells (TNBCs) from metformin induced apoptosis [141].

Metformin treatment in MCF7 cells reportedly caused a 30% reduction in global protein synthesis, which was associated with the AMPK dependent inhibition of the mTOR pathway [107]. In MDA-MB-231 cells lacking LKB1, an upstream kinase that phosphorylates AMPK, and in TSC2 null embryonic fibroblasts of mouse origin, treatment with metformin did not have any effect, indicative of the fact that the mechanism of action of metformin should be dependent on LKB1 and TSC2 [107]. In MCF7 cells, treatment with metformin induced oxidative stress, AMPK and FOXO3a mediated cell death and cell cycle arrest [142]. In breast carcinoma cells, metformin treatment modulated the expression and function of tumor suppressor p53 and reduced the levels of cyclin-D1 thereby causing cell cycle arrest and inhibiting tumor cell growth [142,143]. In TNBCs, treatment with metformin increased the levels phosphorylated-AMPK and induced PARP cleavage in a dose- and time-dependent manner, reduced the levels of phosphorylated EGFR, total EGFR, phosphorylated-MAPK, phosphorylated-Src, cyclin D1, and cyclin E, thus causing and inhibition cell proliferation (partial S phase arrest), colony formation, and promoting apoptosis [144]. In nude mice bearing tumor xenografts of the MDA-MB-231 TNBC cells, metformin treatment significantly reduced tumor growth and cell proliferation when compared to untreated controls [144]. Furthermore, a significant decrease in tumor growth and occurrence was observed in the nude mice, which were pre-treated with metformin prior to the administration of MDA-MB-231 cells [144]. Metformin treatment also inhibited several key enzymes associated with glucose metabolism in MDA-MB-468 TNBC cells, thus indicating that metformin maybe efficient in the treatment of TNBCs [145]. In an in vivo model of metastatic breast cancer, metformin treatment mediated downregulation of platelet-derived growth factor B (PDGF-B) inhibited angiogenesis and the formation of immature vasculature and thus contributed the inhibition of metastasis and further sensitized the metastatic breast cancers to chemotherapy [146].

In MCF7 breast cancer cells, uncoupled reactions accounted for a major fraction of the cellular respiration [147]. Metformin treatment caused a dose-dependent decrease in mitochondrial respiration in the MCF7 cells and led to an upregulation of glycolysis and reduction in cell proliferation [147]. While metformin treatment was associated with inhibition of cell growth in six different basal cancer cells (MDA-MB-468, HCC70, HCC1806, MDA-MB-231, BT20, and HCC1937), the MDA-MB-468 and HCC70 cells were most sensitive [134]. Additionally, a significant reduction in tyrosine and serine phosphorylation of STAT3 was observed in metformin-treated MDA-MB-468, HCC70, MDA-MB-231, and BT20 cells [134]. While overexpression of constitutively active STAT3 negatively affected the efficacy of metformin in these cells, knocking down STAT3 enhanced metformin induced apoptosis [134]. Sequential modulation of DICER and c-Myc played a critical role in metformin mediated anti-cancer effects in MCF7 and BT474 breast cancer cells [105]. In fact, metformin modulated the c-Myc levels in breast cancer cells through its action on miRNA33a, an effect which was reportedly abolished in DICER knockdown SUM159PT cells [105].

## 4. Clinical Data and Trials

Diabetic subjects on metformin were reported to have a significantly lower risk of developing breast cancer when compared to diabetic individuals who were not using metformin to control their blood glucose levels [148,149]. A lower incidence of invasive breast cancer was observed in diabetic patients taking metformin when compared to those on other anti-diabetic (such as sulfonylureas) treatment plans [150]. This correlation is, however, independent of diabetes and thus supports the use of metformin in non-diabetic individuals for cancer prevention. On the other hand, high levels of circulating insulin as well using insulin and insulin analogues to control blood glucose levels were associated to an increased risk of breast cancer [149,151]. The decrease in blood glucose and insulin levels as a result of metformin administration reduced the proliferation of cancer cells and suppressed the growth of the tumor [149,151]. The metformin treatment-associated decrease in circulating hormone (estrogen) levels further supports a reduction in breast cancer incidence among metformin-administered diabetic subjects [149,152,153]. Since breast cancer cells is addicted to glucose utilization via aerobic glycolysis for growth and proliferation, metformin treatment-related increase in energy stress and decrease in glucose levels should also contribute to the suppression of breast cancer growth [149]. Additionally, the reduction in the mitochondrial ATP production as a result of complex I inhibition by metformin further makes the cancer cells susceptible to metformin treatment [47].

A large study involving 2529 breast cancer patients showed a ‘complete response’, with no traces of cancerous cells in the affected breasts or lymph nodes, in diabetic breast cancer patients receiving a combination of chemotherapy and metformin [154]. Additionally, an improved pathologic complete response rate was observed in diabetic breast cancer patients who received both metformin and neoadjuvant chemotherapy than the diabetic patients who received only chemotherapy [154]. The phase 3 adjuvant lapatinib and/or trastuzumab treatment optimization (ALTTO) randomized trial reported that, while insulin administration supported cancer progression and was associated with detrimental effects, metformin-treated HER2 positive diabetic breast cancer patients presented with improved disease-free survival and overall survival rates when compared to HER2 positive diabetic breast cancer patients not on metformin treatment [155]. Although no significant association between exposure to metformin and the occurrence of breast cancer was reported, reports suggest that metformin may improve survival in breast cancer patients with diabetes since metformin administration to breast cancer subjects with diabetes reportedly led to a 45% risk reduction for all-cause mortality [156].

Data from ClincalTrials.gov and cancer.gov showed a list of 44 (search performed on 11 November 2019; Table 1) metformin administration-based clinical trials in breast cancer. While 12 out of the 44 metformin and breast cancer related clinical trials were terminated, withdrawn, or the status remains unknown, 14 clinical trials (out of the 44) were completed, 11 (of the completed 14) of which are specifically related to breast cancer (Table 2), while 3 (of the completed 14) include studies related to breast cancer and other solid tumors of the lung, kidney, liver, and endometrium. From the 11 completed metformin and breast cancer specific clinical trials, only 6 have reported the data or published articles related to the clinical trial. Eighteen breast cancer related clinical trials are currently active, out of which 14 are active and recruiting patients while 4 clinical trials have not started recruiting subjects for the study. Metformin is used as the sole or one of the interventions in nine (Table 2) out of the 14 active and recruiting clinical trials that are specific for breast cancer.

A higher incidence of post-menopausal breast cancer and deteriorating prognosis was observed in obese women when compared to post-menopausal women with normal body mass index (BMI) [157]. Both insulin resistance and the levels of estrogens (both endogenous and exogenous) contribute largely to the effect of obesity on breast cancer risk [5,157]. Exemestane, an irreversible aromatase inhibitor, decreases estrogen levels and is used routinely in the treatment of hormone receptor positive breast cancer [157]. While metformin reduces blood glucose levels by decreasing hepatic gluconeogenesis and glycogenolysis, increasing utilization of glucose of muscles, and improves insulin sensitivity, rosiglitazone improves insulin sensitivity via the activation of the PPARγ receptors [157]. A phase I dose escalation study (NCT00933309, Table 2) assessing the tolerability and pharmacokinetics of exemestane (25 mg/day, orally) in combination with metformin (dose level 1: 1500 mg/day, dose level 2: 2000 mg/day) and rosiglitazone (dose level 1: 6 mg/day, dose level 2: 8 mg/day) in post-menopausal overweight or obese women with hormone receptor positive metastatic breast cancer reported that the oral administration of exemestane with a combination of metformin and rosiglitazone was well tolerated [157]. Administration of metformin (1500 mg, daily, NCT00930579, phase II, Table 2) to 35 non-diabetic overweight women (BMI ≥ 25 kg/m^2^) with breast cancer (stages 0-III) showed no significant difference in the tumor proliferation index (Ki67) when compared to age, BMI, and stage matched historic controls, despite a significant reduction in BMI, cholesterol, and leptin levels in the metformin-treated subjects [158]. Increase in the levels of Raptor, C-Raf, Cyclin B1, Cyclin D1, TRFC, and Syk; while reduction in the levels of pMAPK^pT202, Y204^, JNK^pT183, pT185^, Bad^pS112^, PKCα^pS657^, and Src^pY416^ was observed in the metformin administered (1500 mg, daily, NCT00930579, phase II, Table 2) non-diabetic overweight women with breast cancer when compared to age, BMI, and stage-matched historic controls [159]. This is indicative of the fact that in a clinical setting, metformin administration can influence cancer cell apoptosis, cell cycle, cell signaling, and invasion [159]. A phase II, single arm, ‘window of opportunity’ neoadjuvant metformin clinical study (NCT00897884, phase II, Table 2) conducted in non-diabetic early stage breast cancer patients investigated whether taking metformin (500 mg; thrice daily for ≥ 2 weeks after diagnostic biopsy) until surgery could reduce cell proliferation rates in the tumor tissue and reported reduced levels of phosphorylated Akt and ERK1/2, coupled to reduction on the levels of insulin and insulin receptors indicating that the anti-cancer effect of metformin presents insulin-dependent effects in a clinical scenario [136]. Interestingly, the levels of phospho-AMPK and phospho-ACC levels decreased in post-metformin treated breast tissues [136]. The metformin transporter OCT1 was found to be expressed in all the tissues examined [136]. A clinical trial (NCT01266486, phase II, Table 2) integrated dynamic positron emission tomography, metabolomics, and transcriptomics in breast cancer patients to study the effects of metformin treatment (500 mg for days 1–3, 1000 mg for days 4–6, and 1500 mg thereafter, minimum of 13 days and maximum of 21 days) and reported that metformin administration increases 2-deoxy-2-(^18^F)-fluoro-d-glucose (18FDG) influx into the tumors and activates genes related to mitochondrial metabolism [160]. Although no significant correlation was observed between baseline OCT1 levels and tumor metformin levels, it was interesting that the patient with highest tumor metformin levels correlated to the overexpression of tumor OCT1 [160]. A higher proliferation score was observed in tumors with upregulated genes related to oxidative phosphorylation [160]. The anti-tumor effect of metformin in breast cancer affects breast cancer metabolism and links these anti-cancer effects of metformin to mitochondrial metabolism thereby affecting cell proliferation at a transcriptional level [160]. A phase II clinical trial (NCT01589367, phase II, Table 2), compared the efficacy of a combination of letrozole (2.5 mg/day) and metformin (1000 mg/day for week 1, 1500 mg/day for week 2, 2000 mg/day for week 3) vs. letrozole (2.5 mg/day) and placebo, in ER-positive breast cancer patients [161]. A phase II (NCT01310231, phase II, Table 2) trial showed no significant association between the use of metformin and the stage of breast cancer, characteristics of the tumor at diagnosis, and survival of patients [162,163].

## 5. Monotherapy vs. Combination Therapy

The use of surgery, chemotherapy, and radiation therapy have become the norm for the treatment for most cancers with considerable success in suppressing tumor progression and improving the survival and quality of life of the affected individuals [164]. The hurdle, however, lies in the fact that the use of a single treatment modality/monotherapy approach leads to toxicity and the evolution of resistance, metastasis, and relapse of the disease. Much of the research in the field of oncology is now focused on overcoming these obstacles by treating cancers with a combination of one or more drugs or therapeutic modalities, which was found to be advantageous over monotherapies due to a number of reasons [137,164]. Using targeted therapeutic combination regimens translates to (1) higher efficacy of the treatment and improved overall outcome and prognosis, with decreased incidence of metastasis and relapse; (2) synergistic effects of the drugs, therefore requiring combination drugs to be used at much lower dosage but with maximum efficiency; (3) lesser drug induced toxicity and adverse side effects as a result of lower dosage of the drugs when used in combination; and (4) avoidance of resistance development against the drugs when used in combination [165]. A sound understanding regarding drug interactions and contraindications is necessary to formulate an appropriate drug combination to achieve therapeutic efficiency.

Combinations of two or more chemotherapeutic drug are currently being clinically tested for their efficacy in the treatment of various cancers with satisfactory outcomes [164]. Interest has peaked in combination therapeutic regimens, which employ a chemotherapeutic drug (hormone modulating drugs, anti-metabolite drugs, antibiotics, drugs targeting the structure and function of DNA, drugs that modulate protein translation) or natural compounds/phytochemicals in combination with metformin since it targets cancer cell metabolism and causes energy stress with minimal toxicity, both as a neoadjuvant and adjuvant therapeutic modalities. The feasibility of using of using metformin as one of the drugs in the combination is credited to the multifaceted anti-cancer/anti-tumor ability of metformin in targeting different molecular mechanisms that disrupt the growth and progression of cancers [164]. Several studies have used combination therapy approaches using metformin and phytochemicals/other chemotherapeutic drugs, which have been provided in Table 3.

## 6. Challenges, Future Perspective, and Directions

### 6.1. Mixed Messages and Challenges

The number of epidemiological and meta-analysis studies that support the efficacy of metformin as an anti-cancer/anti-tumor agent have significantly increased ever since diabetologists reported the unusually low rate of cancers among diabetic patients treated with metformin. It was and is still being followed by attempts to provide well defined scientific evidences through lab-based and clinical studies to repurpose metformin and establish it as a drug that can be used in the treatment of wide variety of cancers, including breast cancer. While the identification of new and safer anti-cancer agents is always welcome in the medical field, metformin has garnered a special interest and significance in this regard since it is well tolerated and safe, off patent regulations, easy to synthesize, and can be made available cheaply and extensively in the global market.

In spite of all the studies and data that supports the use of metformin as an anti-cancer agent in breast cancer, certain studies have failed to show any significant association between the use of metformin and the stage of breast cancer, characteristics of the tumor at diagnosis, and survival of patients [162,163]. Patients receiving concurrent metformin and radiation experienced increased locoregional toxicity, higher frequency of treatment breaks, and desquamation/dermatitis despite the fact that several preclinical studies have demonstrated the metformin treatment-associated sensitization to radiation therapy [198,199,200]. Reports have also identified time-related biases in the observational studies that systematically tend to exaggerate the reported anti-tumor effects of metformin [201,202].

Several in vitro and in vivo studies have come up with significant data supporting the use of metformin as an anti-cancer agent in breast cancer, as a monotherapy, and, with even better efficacy when used in combination with other routinely used chemotherapeutic drugs/radiation therapy and or other naturally occurring compounds with known anti-cancer potential. Surprisingly, although several clinical trials (44 breast cancer and metformin treatment-related trials, as discussed above) have been undertaken to provide clinical evidence-based support to the lab-based studies, out of the 14 that have been completed, only 6 have reported the data and published the results. The outcomes of ongoing clinical trials should, in future, provide more concrete answers with regards to the use and efficacy of metformin, in the prevention and treatment of breast cancer in diabetic patients, as well as its safety when used in non-diabetic breast cancer subjects [149].

There are several challenges that ‘metformin’ should face and overcome before it can be universally accepted for its anti-cancer potential, in a manner it is currently accepted and used as an anti-diabetic drug. Dosage of metformin administration as a monotherapy or in combination with other drugs or therapeutic modalities is critical to achieve therapeutic efficiency in the treatment with minimal side effects. When used orally in the treatment of diabetes, the anti-hyperglycemic effects of metformin have been reported at plasma concentrations ranging from around from 10–100 μM [68,137,203]. Interestingly, epidemiological, observational, and meta-analysis reports suggest that oral administration of metformin (normal initial oral dose for; ‘immediate release’ is 500 mg twice/day or 850 mg once/day, with 500 mg increments weekly as tolerated, maximum dose of 2550 mg/day; ‘extended release’ is 500 to 1000 mg once/day, with 500 mg increments weekly as tolerated, maximum dose of 2000 mg/day) in type 2 diabetic patients significantly reduced the risk of many different cancers when compared to diabetic patients on other anti-hyperglycemic treatment regimens [35,37,204,205]. On the other hand, some studies have exercised caution while interpreting observational studies and systematic reviews, suggesting that factors such as the time-related bias were not considered leading to the inference that metformin treatment led to significant (ranging from 20% to 94%) reduction in the risk/incidence of cancer [201,206]. Metformin use showed no effect on the incidence of cancers in studies that avoided these biases [201,206]. In the majority of in vitro/cancer cell based experiments, however, metformin treatment-related inhibitory effect on tumor cell proliferation, activation of apoptotic cell death, and inhibition of tumor progression was only observed at >2–5 mM concentrations of metformin [68,137,138,203,207]. Further studies are warranted to provide a clear insight into the possible dose-dependent effect of metformin on cancer prevention/risk reduction in ‘diabetic’ patients and cancer treatment in ‘diabetic-cancer’ patients. It may be, however, necessary to manage diabetes among cancer patients since the efficacy of metformin as an anti-cancer agent is largely dependent on the concentration of glucose in the tumor microenvironment [5,149]. Pharmacological deprivation of glucose/inhibition of energy deriving glucose metabolism in cancers when combined with metformin treatment should be more beneficial in cancer treatment, thereby eliciting an improved patient response to the therapeutic intervention [5,149].

Additionally, it is still unclear how these massive concentrations can be achieved in a clinical setting when used in the treatment of cancer and whether there would be dosage dependent toxicity related adverse effects that may outweigh the beneficial effect of metformin as an anticancer agent. Although few studies on the metformin transporters, OCTs, PMAT, and MATEs, provide insight into the possible intracellular accumulation of metformin in cancer cells, more studies are warranted in this area to ascertain the specificity to cancer cells and whether the normal non-cancerous cells will be spared from these effects of metformin depending on the absence or presence or the ratio of the metformin intake or extrusion transporters. Additionally, the heterogeneity of the transporter expression in different cancers must be carefully studied and addressed in this regard to arrive at a safe and effective dosage of metformin when used in the treatment of cancer. Studies using metformin in combination with other chemotherapeutic drugs or natural compounds with anti-cancer potential may hold the key to identifying synergistic effects of the combination therapy, thereby effectively decreasing the dosage required with better therapeutic efficiency when compared to usage as a monotherapy in the treatment of cancer.

It is true that several cell-based and in vivo studies have shown the several different molecular mechanisms that support the anti-tumor potential of metformin monotherapy. Such studies are indeed very important to elucidate molecular mechanisms and identify therapeutic targets of metformin. In clinical trials (Table 2), however, metformin is rarely studied as the sole therapeutic agent, but is used as co-treatment with other routinely used chemotherapeutic drugs [208]. This is indicative of the fact that it is unlikely, although not impossible, that metformin will gain importance as a monotherapy in cancer treatment. Nonetheless, metformin surely has the potential as an anti-cancer drug, which can be accepted and be used as neoadjuvant/adjuvant therapy or as co-treatment in combination with routine anti-cancer treatment regimens [208].

A vast number of studies suggest that metformin co-treatment can overcome resistance to chemotherapeutic drugs/radiation and can sensitize the cancer cells to the ant-cancer agents/radiation. However, like any other chemotherapeutic drug/treatment modality, long-term metformin administration can also be challenged with the evolution of treatment-related resistance. In response to long-term metformin treatment, MCF7 breast cancer cells reportedly developed cross-resistance to metformin and tamoxifen and was dependent on the constitutive activation of Akt/Snail1/E-cadherin signaling [209]. As discussed earlier in the article, combinatorial therapeutic approaches are key to avoiding treatment-related drug resistance.

### 6.2. Metformin and Breast Cancer Biomarkers

In cancers, identification of suitable diagnostic and prognostic biomarkers is critical to screening for, early detection of, development of therapeutic strategy for, and evaluating the responsiveness to the therapeutic intervention of the disease. ER and PR hormone receptors, BRCA1 and BRCA2 gene mutations, HER2/neu gene amplification or protein overexpression, plasminogen activator inhibitor 1 (PAI-1), urokinase-type plasminogen activator (uPA), 70-gene signature (Mammaprint^®^), and 21-gene signature (Oncotype DX^®^) are being currently used as markers for breast cancers [5,210,211]. More recently, the cancer testis antigens (CTAs) that are in physiology normally expressed in the testis and placenta are expressed in different forms of cancers and have been implicated in tumorigenesis, thus gaining importance as diagnostic molecular signatures with prognostic value in cancers including breast cancers [5,212,213]. CTAs are involved in cellular proliferation, migration/motility, colonization, and cell division in normal germ cells, while in cancer cells, CTAs have been implicated in sustenance of growth, maintaining nutrient and oxygen supply through the activation of angiogenesis, evading apoptotic cell death, increase in tissue invasiveness and metastasis, and development of resistance to anti-cancer drugs [214]. In breast cancer, CTAs, such as MAGE-1, AKAP4, NY-BR-1, CTAG1, BAGE1, MAGE-A10, SP17, NY-ESO-1, and MAGE-A, were found to play important roles in activating cell proliferation, inhibition of apoptosis, and promoting tissue invasion and metastasis [5,215,216,217,218,219,220,221,222]. The CTA, AKAP4, in particular seems to be an interesting candidate for a serum-based diagnostic test for the early detection and diagnosis of breast cancer [221]. SP17, MAGE-A, NY-ESO-1, and MAGE-A10 on the other hand were more specifically associated with TNBCs [216,218,219]. There is but little evidence that could link the occurrence of diabetes to the expression of the CTAs, which can then be possibly used as diagnostic markers to link diabetes and the incidence of breast cancer or even a specific kind of breast cancer. Moreover, it is also necessary to evaluate if there is any association between the anti-hyperglycemic treatment used in diabetes, such as insulin or metformin, and the expression or levels of specific CTAs in breast tissue, which could ultimately also serve as prognostic markers to study the response of the cancer to metformin intervention.

### 6.3. Efficacy in Non-Diabetic Patients and Non-Diabetic Cancer Patients

As discussed, several observational studies, systematic reviews, and meta-analysis studies report the ability and efficacy of metformin in risk reduction of many types of cancer, including breast cancer, in type 2 diabetic patients on a metformin treatment regimen. Moreover, women using metformin in combination with other anti-hyperglycemic drugs reportedly showed a lower risk of breast cancer occurrence, although not significant, when compared to women using metformin alone [223]. While metformin notably inhibits cell growth and proliferation-related pathways, these effects may be in part related to the ability of metformin to reduce insulin resistance, insulin levels, and circulating levels of glucose [65]. However, it remains largely unknown if non-diabetic women with breast cancer, or non-diabetic individuals with other forms of cancer, would benefit from taking metformin [149].

Medical science has advanced in leaps and bounds in the area of cancer treatment, with research teams constantly searching for and introducing newer and more efficient drugs/treatment modalities for the treatment of cancer. Nonetheless, in a world where cancer is one of the most feared and dreaded diseases, it will depend on the results of the currently active clinical trials to determine and recommend whether it is feasible and beneficial to administer metformin (1) as a cancer ‘preventive’ drug in non-diabetics to reduce the risk of cancers or (2) as an intervention, either alone or in combination with other anti-cancer drugs, for the treatment of cancer ‘non-diabetic’ cancer patients [149,224]. While using metformin as a preventive drug may decrease the occurrence of cancers in non-diabetic individuals, in non-diabetic cancer patients, metformin should increase the efficacy of anti-cancer drugs and show improved prognosis.

### 6.4. Future Directions—Metformin and a Proposed Path Towards Precision Medicine

Metformin has gained global attention for its potential as an anti-cancer drug and is currently being tested in in vitro cell-based assays, in vivo animal experiments, and in clinics at many cancer research facilities around the world. Although the end results are ‘connected’ in terms of molecular mechanism of action, inhibition of cell proliferation, activation of cancer cell death, and tumor suppression, there seems to be ‘disconnect’ when it finally comes from the bench to bedside application. It is true that targeting a unified mechanism makes it easier to treat a disease. However, the heterogeneity and variation in diseases such as cancer calls for more ‘personalized’ and ‘precise’ case-by-case approach in treatment modalities for a better outcome.

**Figure 3:**
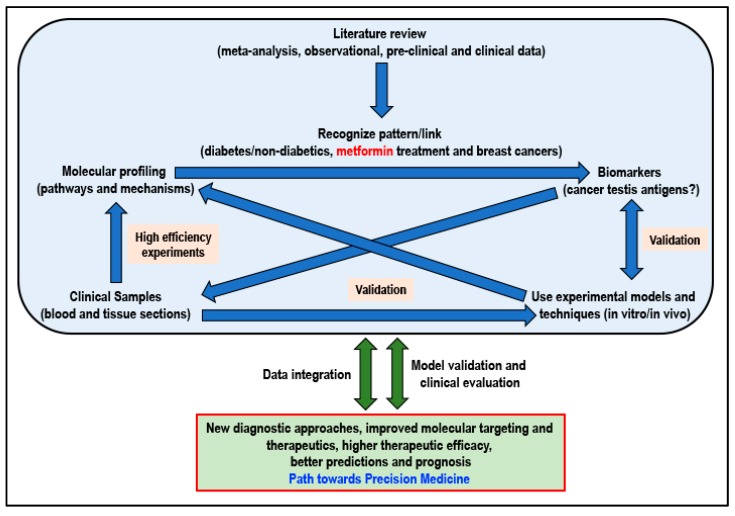
Proposed workflow for better integration of currently available and new results/data for a path towards personalized precision medicine (Idea adapted from Podo F et al., 2010) [225].

Not ignoring the fact that basic biomedical research will always remain extremely important, research resources must be channeled into this area of precision medicine that will be tailored to fit a patient based on the risk of disease and predicted response to the therapeutic intervention. When considering metformin for its potential cancer preventive or cancer-treatment effect, adhering to an approach of precision medicine and adopting a workflow (Figure 3) that will integrate information, knowledge, skills, and research data at all levels of research will certainly be beneficial to ultimately determine if the answer to cancer can indeed be found in a flower.

## Figures and Tables

**Table 1:** Status of metformin administration related clinical trials in various cancers (https://clinicaltrials.gov/).

Serial No:.	Type of Cancer	Total Number of Registered Trials	Completed	Active, not Recruiting	Active, Recruiting	Terminated	Withdrawn	Unknown Status #
1	Breast Cancers	44	14	4	14	5	1	6
2	Prostate Cancers	27	6	5	9	2	4	1
3	Colorectal Cancers	20	4	3	7	6	0	0
4	Lung Cancers	19	4	6	3	5	0	1
5	Oral Cancers	5	0	2	3	0	0	0
6	Head & Neck Cancers	9	1	3	3	2	0	0

# Unknown Status: The study has crossed its proposed date of completion, but status remains unverified for over 2 years.

**Table 2:** Metformin and completed/ongoing (active and recruiting) clinical trials specific for breast cancers (https://www.cancer.gov/about-cancer/treatment/clinical-trials/intervention/metformin-hydrochloride and https://clinicaltrials.gov/).

Serial No:	Name/ID	Trial Phase	Intervention Using Metformin	Objectives	Type of Cancer	Clinicaltrials.Gov ID (NCT Number)/Status Completion Year or Estimated Primary Completion Year	Publications/References
1	Clinical and Biologic Effects of Metformin in Early Stage Breast Cancer	Phase II	Metformin	To determine if taking metformin prior to surgery can reduce cell proliferation rates in tumor tissue	Breast Cancer	NCT00897884/Completed July 2011	[136]
2	Effect of Metformin on Breast Cancer Metabolism	Phase II	Metformin	Measure metformin induced effects in phosphorylation of S6K, 4E-BP-1 and AMPK via immunohistochemical analysis	Breast Cancer	NCT01266486/Completed May 2014	[160]
3	Metformin in Breast Cancer, Visualized with Positron Emission Tomography	Phase I	Radiation: 11C-metformin	Metformin uptake in breast cancer	Breast Cancer	NCT02882581/Completed Oct 2017	No results posted
4	A Trial of Standard Chemotherapy with Metformin (vs Placebo) in Women with Metastatic Breast Cancer	Phase II	Metformin + standard chemotherapy (containing anthracyclines, platinum, taxanes or capecitabine) vs. placebo + standard chemotherapy	Progression free survival	Metastatic Breast Cancer	NCT01310231/Completed Mar 2018	[162,163]
5	Study of Erlotinib and Metformin in Triple Negative Breast Cancer	Phase I	Erlotinib + Metformin	The maximum tolerated dose of metformin in combination with a fixed dose of 150 mg erlotinib daily	Breast Cancer	NCT01650506/Completed June 2016	No results posted
6	Neoadjuvant Letrozole Plus Metformin vs Letrozole Plus Placebo for ER-positive Postmenopausal Breast Cancer	Phase II	Letrozole + Metformin vs. Letrozole + Placebo	Clinical response rate	Hormone Receptor Positive Malignant Neoplasm of Breast	NCT01589367/Completed Aug 2018	[161]
7	Metformin Hydrochloride vs. Placebo in Overweight or Obese Patients at Elevated Risk for Breast Cancer	Phase I (Early)	Metformin vs. Placebo	Changes in the phosphorylation of proteins after metformin exposure	Breast Cancer and Obesity	NCT01793948/Completed Jan 2018	No results posted
8	Efficacy and Safety of Adjuvant Metformin for Operable Breast Cancer Patients	Phase II	Metformin (500 mg/1000 mg) vs. Placebo	Weight loss	Breast Cancer	NCT00909506/Completed Dec 2011	No results posted
9	Myocet + Cyclophosphamide + Metformin vs. Myocet + Cyclophosphamide in 1st Line Treatment of HER2 Neg. Metastatic Breast Cancer Patients	Phase II	Metformin + Myocet + Cyclophosphamide vs. Myocet + Cyclophosphamide	Progression-free survival	Human Epidermal Growth Factor 2 Negative Carcinoma of Breast	NCT01885013/Completed May 2015	No results posted
10	The Impact of Obesity and Obesity Treatments on Breast Cancer	Phase I	Exemestane vs. Exemestane + Avandamet (Metformin + Rosiglitazone)	Dose-limiting toxicity	Breast Cancer	NCT00933309/Completed Aug 2012	[157]
11	Metformin Pre-surgical Pilot Study	Phase II	Metformin	Effects of metformin on AMPK/mTOR signaling pathway	Breast Cancer	NCT00930579/Completed Oct 2011	[158,159]
12	I-SPY 2 TRIAL: Neoadjuvant and Personalized Adaptive Novel Agents to Treat Breast Cancer	Phase II	AMG 479 (Ganitumab) + Metformin	Comparing the efficacy of novel drugs in combination with standard chemotherapy with the efficacy of standard therapy alone and identification of improved treatment regimens for subjects on the basis of molecular characteristics (biomarker signatures) pertaining to their disease	Breast Neoplasms/Cancer/Tumors	NCT01042379/Active-Recruiting Dec 2020	No results posted
13	Randomized Trial of Neo-adjuvant Chemotherapy with or without Metformin for HER2 Positive Operable Breast Cancer	Phase II	Chemotherapy (Taxotere, Carboplatin, Herceptin + Pertuzumab) vs. Chemotherapy + Metformin	Pathologic complete response	HER2-positive Breast Cancer	NCT03238495/Active-Recruiting Sep 2019	No results posted
14	Pre-Surgical Trial of the Combination of Metformin and Atorvastatin in Newly Diagnosed Operable Breast Cancer	Phase I (Early)	Metformin + Atorvastatin (pre-treatment, prior to breast surgery)	Change in the tissue levels of the proliferation marker Ki-67. Tumor proliferation as measured by the natural expression of Ki.67 staining of breast cancer cells	Breast Cancer, Breast Tumors, Cancer of Breast	NCT01980823/Active-Recruiting Dec 2021	No results posted
15	Metformin Hydrochloride in Preventing Breast Cancer in Patients with Atypical Hyperplasia or In Situ Breast Cancer	Phase III	Metformin vs. Placebo	Test for the presence or absence of cytological atypia in unilateral or bilateral RPFNA aspirates after 12 and 24 months	Atypical Ductal Breast Hyperplasia, BRCA1 Mutation Carrier, BRCA2 Mutation Carrier, Ductal Breast Carcinoma in Situ, Lobular Breast Carcinoma in Situ	NCT01905046/Active-Recruiting Jun 2022	No results posted
16	NeoMET Study in Neoadjuvant Treatment of Breast Cancer	Phase II	Metformin + chemotherapy (docetaxel + epirubicin + cyclophosphamide) vs. chemotherapy	Pathologic complete response rate	Breast Cancer	NCT01929811/Active-Recruiting Sep 2021	No results posted
17	Neoadjuvant FDC with Melatonin or Metformin for Locally Advanced Breast Cancer (MBC1)	Phase II	Metformin + chemotherapy (fluoruracil + doxorubicin + cyclophosphamide) vs. Melatonin + chemotherapy vs. Chemotherapy	Response rate and pathomorphological response	Breast Cancer	NCT02506777/Active-Recruiting Aug 2020	No results posted
18	Neoadjuvant Toremifene with Melatonin or Metformin for Locally Advanced Breast Cancer (MBC1	Phase II	Metformin + toremifene vs. Melatonin + toremifene vs. Toremifene	Response rate and pathomorphological response	Breast Cancer	NCT02506790/Active-Recruiting Aug 2020	No results posted
19	Metformin Hydrochloride and Doxycycline in Treating Patients with Localized Breast or Uterine Cancer	Phase II	Metformin + doxycycline vs. Doxycycline	Change in the percent of stromal cells expressing Caveolin-1 (Cav1) at an intensity of 1+ or greater as assessed by immunohistochemistry	Breast Carcinoma	NCT02874430/Active-Recruiting Feb 2021	No results posted
20	Evaluation of the effect of Metformin on Metastatic Breast Cancer as Adjuvant Treatment	Phase I	Metformin	Disease progression through tumor size	Metastatic Breast Cancer	NCT04143282/Active-Recruiting Dec 2019	No results posted

**Table 3:** Metformin-based combinatorial therapy in the treatment of breast cancers.

Conventional Chemotherapeutic Drug or Treatment Modality	Effect and Possible Mechanism of Action	Cells/in vivo Model Used	Ref.
2-Deoxyglucose (2DG)	Improved the efficacy of sodium-iodide symporter-mediated targeted radioiodine therapy breast cancer cells.	MCF7, MDA-MB-231	[166]
	Inhibited decreased bioenergetic metabolism and decreased viability in feline mammary carcinoma cells.	AlRB (HER2^+++ve^), AlRATN (HER2-^ve^)	[167]
	Induced AMPK dependent detachment and decrease in proliferation of viable breast cancer cells in vitro.	MCF7, MDA-MB-231	[168]
	Significantly reduced cell viability and increased PARP cleavage associated apoptosis.	MDA-MB-231, HCC1806	[169]
	Reversed multidrug resistance, increased doxorubicin (DOX) accumulation, resumed p53 function via inhibition of MDM2 and MDM4 leading to G2/M cell cycle arrest and apoptosis, inhibited glucose uptake, production of lactate, fatty acid and ATP and downregulated the Akt/mTOR pathway.	MCF7/DOX resistant cells	[170]
5-Fluorouracil, Epirubicin and Cyclophosphamide (FEC)	Metformin synergizes FEC combination therapy via AMPK dependent mechanism in non-stem/parental breast cancer cells, while in cancer stem cells (CSCs) the synergistic effect of the combination treatment was found to be independent of AMPK. In CSCs, while metformin accelerated glucose consumption and lactate production, the production of intracellular ATP was significantly diminished leading to energy stress and impairment of the ability of CSCs to repair the FEC induced DNA damage.	MCF7, MDA-MB-231, MDA-MB-468, HCC1937, SKBR3, T47D, MCF10A, MRC-5 (human embryonic lung fibroblasts), breast CSCs	[171]
Aspirin	Aspirin and metformin in combination synergistically activated apoptotic cancer cell death in vitro and reduced tumor growth in vivo facilitated by enhancing the secretion of TGFβ1. Reducing the estrogen levels in circulation or its inhibition maximized the anti-tumor activity of the combinatorial drug.	4T1, BALB/c mice inoculated with 4T1 cells	[172]
	Metformin treatment alone altered morphology decreased viability and migration of ER+ve MCF7 cells. The combination of aspirin and metformin synergistically altered morphology decreased viability and migration in TNBC MDA-MB-231 cells. HER2+ve SK-BR-3 cells showed a partial response to monotherapy (aspirin or metformin) and combinatorial therapy (aspirin and metformin).	MCF7, SK-BR-3, MDA-MB-231,	[173]
Chrysin	Synergistic growth inhibitory effects due to suppression of hTERT and cyclin D1 gene expression	T47D	[174]
Curcumin	Inhibition of tumor proliferation and growth associated with reduced VEGF expression and angiogenesis, induction of p53 independent apoptosis, and activation of Th2 related immune response with no toxicity.	EMT6/P cells, BALB/c mice inoculated with EMT6/P cells	[175]
	Combination of PEGylated PLGA nanoparticle co-encapsulated metformin and curcumin exhibited dosage dependent toxicity and synergistic antiproliferative effect causing significant cell cycle/growth arrest in the cancer cells. The hTERT gene expression was significantly inhibited in cells treated with the nano-formulation of metformin-curcumin when compared to delivery of either metformin or curcumin alone.	T47D	[176]
Denosumab	BRCA1 haplo-insufficiency driven RANKL gene overexpression was hampered by metformin treatment and disrupted the RANKL mediated auto-regulatory feedback in CSCs thereby sensitizing the CSC to denosumab and synergistically reducing the cancer initiating cell population and their capacity for self-renewal.	Breast CSCs, MDA-MB-436	[177]
Dichloroacetic Acid (DCA)	DCA and metformin when used in combination synergistically induced caspase dependent apoptosis in cancer cells. Metformin-associated oxidative stress-induced damage was amplified by DCA treatment associated pyruvate dehydrogenase kinase 1 inhibition thereby reducing metformin mediated lactate production.	MCF7, T47D, MCF10A	[178]
	The combinatorial therapeutic strategy using DCA and metformin inhibited key glycolytic enzymes—hexokinase 2, lactate dehydrogenase A, and enolase 1. An activation of HIF1α abolished the effect of the combination therapy and reversed the inhibition on the expression of the glycolytic enzymes and reduced cell death.	MCF7, H1299, HDF, MCF10A	[179]
Doxorubicin (DOX) + 2-Deoxy-2-(F)-Fluoro-d-Glucose (2FDG)	Metformin treatment increased pAMPK levels while the levels of pAkt and pERK decreased. 2FDG incorporation and phosphorylation increased upon metformin treatment.	MDA-MB-453, MDA-MB-468, SK-BR-3, BT474	[180]
DOX	Nanoparticle co-encapsulated metformin and DOX achieved good tumor penetration, inhibited NF-κB activity, and decreased TNFα and IL6 expressions leading to the significant decrease in cancer cell proliferation. The nano-formulation of metformin and DOX showed a therapeutic effect in the treatment of lipopolysaccharide (LPS)-induced pulmonary metastasis model of murine 4T1 cells.	4T1, BALB/c mice inoculated with 4T1 cells	[181]
	Nanoparticle co-encapsulated metformin and DOX (dual drug loaded) treatment showed increased toxicity and apoptotic cell death in DOX resistant MCF7 cells. The enhanced efficiency and cytotoxicity were attributed the to the intracellular accumulation of the drugs via enhanced cellular uptake and reduction in drug efflux leading to significant energy stress (reduced cellular ATP) and inhibition of multidrug resistance (MDR) mediating P-glycoprotein (P-gp).	MCF7, MCF7/DOX resistant cells	[182]
	Metformin and DOX dual drug-loaded nanoparticles effectively reduced P-gp expression and activity, increased energy stress as evidenced by reduced intracellular ATP levels, and sensitized the cells to DOX induced apoptotic cell death.	MCF7, MCF7/DOX resistant cells	[183]
	Metformin treatment associated AMPK dependent anti-tumor effect was observed in addition to the inhibition of NF-κB and cyclin D1 gene expression. Combinatorial treatment was found to be more effective in decreasing tumor volume and improve overall rate of survival in the animals. Higher rates of apoptosis were observed in histopathological samples derived from animals to which the combination treatment was administered. Metformin treatment-associated reduction in the P-gp expression and elimination of Ki-67 positive cancer cells were observed in MCF7/ADR tumor xenografts.	The Ehrlich ascites carcinoma cells (derived from mouse breast adenocarcinoma cells) were implanted and allowed to multiply in the peritoneal cavity of Swiss albino mice. Solid Ehrlich carcinoma were derived by implanting EAC cells subcutaneously in Swiss albino mice.	[184]
	The combinatorial treatment synergistically reversed DOX resistance both in vitro and in vivo. Metformin inhibited tumor growth. The cytotoxic effects of metformin were enhanced by increasing the levels of ROS while the levels of ATP levels depleted.	MCF7/ADR cells-DOX resistant cells, subcutaneously implanted MCF7/ADR cells in nu/nu mice	[185]
Erlotinib	The combination treatment synergistically induced apoptotic cell death and reduced the phosphorylation of EGFR, Akt, S6, and 4EBP1, prevented colony formation and inhibited mammosphere outgrowth.	MDA-MB-468, MDA-MB-157, MDA-MB-435S, MDA-MB-436, MDA-MB-231, MX-1, MCF7, BT20, L56Br-C1, CAOV-3, HCC1143, HCC1806, HCC1937, HCC1987, HCC70, HCC38, BT549, mice implanted with MDA-MB-468 cells into the mammary fat pad	[186]
Everolimus	Metformin-induced additive effects were observed when used as a co-treatment with everolimus and inhibited cell proliferation and colony formation ability. The additive effect of the combinatorial treatment was also related to the inhibition of mitochondrial respiration and mTOR growth signaling.	MCF7, MDA-MB-231, T47D	[187]
	Inhibition of cell proliferation and tumor growth was observed both in cultures and mouse xenograft models treated with a combination of everolimus and metformin. Significant decrease in the levels of phosphorylated S6 ribosomal protein and 4E-BP1 was observed upon combination treatment.	HCC1428, MDA-MB-468, BT549, BALB/c mice inoculated with HCC1428 cells	[188]
Flavone	Significant inhibition of cell viability, increased apoptosis, decrease in the expression of murine double minute X (MDMX), and activation of p53 via the PI3K/Akt pathway was observed in the combination drutreated cells. Apoptosis was mediated by decrease in Bcl2 and increase in the levels of Bax and caspase 3.	MCF10A, MCF7, MDA-MB-231	[189]
Melatonin	DMBA induced tumor incidence, tumor growth, and volume were reduced by the combination treatment. The apoptotic stimulation observed in the cancer cells was attributed to the activation of caspase 3.	7, 12-dimethylbenz[a]anthracene (DMBA) induced in vivo rat model of breast cancer	[190]
Paclitaxel (PTX)	Co-delivery of PTX and metformin using a folate-modified amphiphilic and biodegradable biomaterial synergistically decreased cell proliferation and induced apoptosis through the toll-like receptor (TLR) signaling via the modulation of the TLR-MyD88-ERK pathway (responsible for tumor growth, progression, metastasis, and drug resistance).	4T1 cells, BALB/c mice inoculated with 4T1 cells	[191]
Silibinin	Synergistic effect on growth inhibition of cancer cells was observed when silibinin and metformin were used in combination. Downregulation of hTERT and cyclin D1 was observed with the combinatorial therapeutic approach.	T47D	[192]
Spautin-1	Deletion of the essential autophagy gene, Rb1cc1, suppressed tumorigenesis in BRCA1-deficient mice, while tumor growth and distribution of histological subtypes were not affected by loss of Rb1cc1. Co-treatment using spautin-1 (autophagy inhibitor) and metformin (mitochondrial complex-1 inhibitor) efficiently reduced the oxidative respiratory capacity, colony forming ability, and tumor growth.	Tumor cells derived from BRCA1-deficient tumors, Rb1cc1^+/+^ brca1^F/F^ trp53^F/F^ K14-Cre mice, Rb1cc1^F/+^ brca1^F/F^ trp53^F/F^ K14-Cre mice and Rb1cc1^F/F^ brca1^F/F^ trp53^F/F^ K14-Cre mice, athymic nude-Foxn1^nu^ mice transplanted with tumor cells derived from BRCA1-deficient tumors	[193]
Tamoxifen	The dosage of tamoxifen required for growth inhibition of cells was much lower when combined with metformin than when used as a monotherapy. The combination treatment inhibited cellular proliferation, DNA replication activity, colony formation, and activated apoptotic cell death in ER+ve breast cancer cells. The involvement of the Bax/Bcl2 apoptotic pathway and the AMPK/mTOR/p70S6 growth pathways were implicated in the beneficial effects of this combinatorial therapy approach.	MCF7, ZR-75-1	[194]
Topotecan	Metformin and topotecan dual drug carrier nanoparticles were found to be synergistically cytotoxic for the breast cancer cells, effectively promoting cell death via mitochondrial membrane depolarization and cell cycle arrest.	MDA-MB-231, 4T1	[195]
Vitamin D3	The combination treatment of metformin and vitamin D3 in synergistically inhibited cell proliferation and activated apoptosis in breast cancer cells. Mechanisms involving activation of AMPK, upregulation of Bax, cleavage of caspase 3, and inhibition of pBcl2, c-Myc, pIGF-IR, pmTOR, pP70S6K, and pS6 were implicated in anti-cancer activity of the combinatorial treatment.	MDA-MB-231	[196]
Radiation	A higher tumor response to radiation was observed in diabetic breast cancer patients who received metformin and partly yielded survival benefits.	Meta-analysis/Clinical	[197]

## Abbreviations

ACC	Acetyl CoA carboxylase
AKAP4	A-kinase anchoring protein 4
AMP	Adenosine monophosphate
AMPK	5′ AMP activated protein kinase
ATP	Adenosine triphosphate
BAGE	B melanoma antigen
CTA	Cancer testis antigen
DDIT4/REDD1	DNA Damage Inducible Transcript 4/Regulated in DNA Damage 1
EMT	Epithelial mesenchymal transition
ER	Estrogen receptor
ERK	Extracellular signal-regulated kinase
ETC	Electron transport chain
HER2	Human epidermal growth factor receptor 2
IGF1	Insulin-like growth factor 1
IRS1	Insulin receptor substrate 1
MAGE	Melanoma antigen
MAPK	Mitogen activated protein kinase
MATE1/2	Multidrug and toxin extrusion protein-1 and 2
mTOR	mammalian target of rapamycin
NF-κB	Nuclear factor kappa-B
OCT1/2/3	Organic cation transporters 1, 2 and 3
PAI-1	Plasminogen activator inhibitor 1
PI3K	Phosphoinositide 3-kinase
PMAT	Plasma membrane monoamine transporter
PR	Progesterone receptor
PTP	Permeability transition pore
SP17	Sperm protein 17
STAT3	Signal transducer and activator of transcription 3
TNBC	Triple negative breast cancer
TNFα	Tumor necrosis factor alpha
TSC2	Tuberous sclerosis 2
UAP1	UDP-N-acetylglucosamine pyrophosphorylase 1
uPA	Urokinase-type plasminogen activator
UPR	Unfolded Protein Response
WHO	World Health Organization

## References

[1] World Health Organization. https://apps.who.int/iris/bitstream/handle/10665/204871/9789241565257_eng.pdf;jsessionid=CB10185391030DF727E80B7DC9747873?sequence=1.

[2] European Society of Cardiology. https://www.escardio.org/Sub-specialty-communities/European-Association-of-Preventive-Cardiology-(EAPC)/News/global-statistics-on-diabetes.

[3] Leon B.M., Maddox T.M. (2015). Diabetes and cardiovascular disease: Epidemiology, biological mechanisms, treatment recommendations and future research. World J. Diabetes.

[4] Min T.Z., Stephens M.W., Kumar P., Chudleigh R.A. (2012). Renal complications of diabetes. Br. Med. Bull..

[5] Samuel S.M., Varghese E., Varghese S., Busselberg D. (2018). Challenges and perspectives in the treatment of diabetes associated breast cancer. Cancer Treat. Rev..

[6] Said G. (2007). Diabetic neuropathy—A review. Nat. Clin. Pr. Neurol..

[7] Blendea M.C., Thompson M.J., Malkani S. (2010). Diabetes and Chronic Liver Disease: Etiology and Pitfalls in Monitoring. Clin. Diabetes.

[8] Boyle P., Boniol M., Koechlin A., Robertson C., Valentini F., Coppens K., Fairley L.L., Boniol M., Zheng T., Zhang Y. (2012). Diabetes and breast cancer risk: A meta-analysis. Br. J. Cancer.

[9] Hardefeldt P.J., Edirimanne S., Eslick G.D. (2012). Diabetes increases the risk of breast cancer: A meta-analysis. Endocr. Relat. Cancer.

[10] Larsson S.C., Mantzoros C.S., Wolk A. (2007). Diabetes mellitus and risk of breast cancer: A meta-analysis. Int. J. Cancer.

[11] Stattin P., Björ O., Ferrari P., Lukanova A., Lenner P., Lindahl B., Hallmans G., Kaaks R. (2007). Prospective Study of Hyperglycemia and Cancer Risk. Diabetes Care.

[12] Giovannucci E., Harlan D.M., Archer M.C., Bergenstal R.M., Gapstur S.M., Habel L.A., Pollak M., Regensteiner J.G., Yee D. (2010). Diabetes and cancer: A consensus report. Diabetes Care.

[13] Bailey C.J. (2017). Metformin: Historical overview. Diabetologia.

[14] Bailey C.J., Day C. (2004). Metformin: Its botanical background. Pract. Diabetes Int..

[15] Leone A., Di Gennaro E., Bruzzese F., Avallone A., Budillon A. (2014). New perspective for an old antidiabetic drug: Metformin as anticancer agent. Cancer Treat. Res..

[16] Misbin R.I. (2004). The Phantom of Lactic Acidosis due to Metformin in Patients With Diabetes. Diabetes Care.

[17] Vecchio S., Protti A. (2011). Metformin-induced lactic acidosis: No one left behind. Crit Care.

[18] Fitzgerald E., Mathieu S., Ball A. (2009). Metformin associated lactic acidosis. BMJ.

[19] Marshall S.M. (2017). 60 years of metformin use: A glance at the past and a look to the future. Diabetologia.

[20] Correia S., Carvalho C., Santos M.S., Seica R., Oliveira C.R., Moreira P.I. (2008). Mechanisms of action of metformin in type 2 diabetes and associated complications: An overview. Mini Rev. Med. Chem..

[21] Nesti L., Natali A. (2017). Metformin effects on the heart and the cardiovascular system: A review of experimental and clinical data. Nutr. Metab. Cardiovasc. Dis..

[22] Iranshahy M., Rezaee R., Karimi G. (2019). Hepatoprotective activity of metformin: A new mission for an old drug?. Eur. J. Pharmacol..

[23] Li Y., Liu L., Wang B., Wang J., Chen D. (2013). Metformin in non-alcoholic fatty liver disease: A systematic review and meta-analysis. Biomed. Rep..

[24] Yanardag R., Ozsoy-Sacan O., Bolkent S., Orak H., Karabulut-Bulan O. (2005). Protective effects of metformin treatment on the liver injury of streptozotocin-diabetic rats. Hum. Exp. Toxicol..

[25] Brackett C.C. (2010). Clarifying metformin’s role and risks in liver dysfunction. J. Am. Pharm. Assoc..

[26] Corremans R., Vervaet B.A., D’Haese P.C., Neven E., Verhulst A. (2018). Metformin: A Candidate Drug for Renal Diseases. Int. J. Mol. Sci..

[27] Rotermund C., Machetanz G., Fitzgerald J.C. (2018). The Therapeutic Potential of Metformin in Neurodegenerative Diseases. Front. Endocrinol..

[28] Ma J., Liu J., Yu H., Chen Y., Wang Q., Xiang L. (2016). Beneficial Effect of Metformin on Nerve Regeneration and Functional Recovery After Sciatic Nerve Crush Injury in Diabetic Rats. Neurochem. Res..

[29] Mao-Ying Q.-L., Kavelaars A., Krukowski K., Huo X.-J., Zhou W., Price T.J., Cleeland C., Heijnen C.J. (2014). The anti-diabetic drug metformin protects against chemotherapy-induced peripheral neuropathy in a mouse model. PLoS ONE.

[30] Bahrambeigi S., Yousefi B., Rahimi M., Shafiei-Irannejad V. (2019). Metformin; an old antidiabetic drug with new potentials in bone disorders. Biomed. Pharmacother..

[31] Prattichizzo F., Giuliani A., Mensà E., Sabbatinelli J., De Nigris V., Rippo M.R., La Sala L., Procopio A.D., Olivieri F., Ceriello A. (2018). Pleiotropic effects of metformin: Shaping the microbiome to manage type 2 diabetes and postpone ageing. Ageing Res. Rev..

[32] Barzilai N., Crandall J.P., Kritchevsky S.B., Espeland M.A. (2016). Metformin as a Tool to Target Aging. Cell Metab..

[33] Novelle M.G., Ali A., Dieguez C., Bernier M., de Cabo R. (2016). Metformin: A Hopeful Promise in Aging Research. Cold Spring Harb. Perspect. Med..

[34] Pryor R., Cabreiro F. (2015). Repurposing metformin: An old drug with new tricks in its binding pockets. Biochem. J..

[35] DeCensi A., Puntoni M., Goodwin P., Cazzaniga M., Gennari A., Bonanni B., Gandini S. (2010). Metformin and Cancer Risk in Diabetic Patients: A Systematic Review and Meta-analysis. Cancer Prev. Res..

[36] Zi F., Zi H., Li Y., He J., Shi Q., Cai Z. (2018). Metformin and cancer: An existing drug for cancer prevention and therapy. Oncol. Lett..

[37] Kim H.J., Lee S., Chun K.H., Jeon J.Y., Han S.J., Kim D.J., Kim Y.S., Woo J.-T., Nam M.-S., Baik S.H. (2018). Metformin reduces the risk of cancer in patients with type 2 diabetes: An analysis based on the Korean National Diabetes Program Cohort. Medicine.

[38] Yao L., Liu M., Huang Y., Wu K., Huang X., Zhao Y., He W., Zhang R. (2019). Metformin Use and Lung Cancer Risk in Diabetic Patients: A Systematic Review and Meta-Analysis. Dis. Markers.

[39] Evans J.M., Donnelly L.A., Emslie-Smith A.M., Alessi D.R., Morris A.D. (2005). Metformin and reduced risk of cancer in diabetic patients. BMJ.

[40] Bowker S.L., Majumdar S.R., Veugelers P., Johnson J.A. (2006). Increased cancer-related mortality for patients with type 2 diabetes who use sulfonylureas or insulin. Diabetes Care.

[41] Li D., Yeung S.C., Hassan M.M., Konopleva M., Abbruzzese J.L. (2009). Antidiabetic therapies affect risk of pancreatic cancer. Gastroenterology.

[42] Currie C.J., Poole C.D., Gale E.A. (2009). The influence of glucose-lowering therapies on cancer risk in type 2 diabetes. Diabetologia.

[43] Bost F., Decoux-Poullot A.G., Tanti J.F., Clavel S. (2016). Energy disruptors: Rising stars in anticancer therapy?. Oncogenesis.

[44] Sośnicki S., Kapral M., Węglarz L. (2016). Molecular targets of metformin antitumor action. Pharmacol. Rep..

[45] Daugan M., Dufaÿ Wojcicki A., d’Hayer B., Boudy V. (2016). Metformin: An anti-diabetic drug to fight cancer. Pharmacol. Res..

[46] Vial G., Detaille D., Guigas B. (2019). Role of Mitochondria in the Mechanism(s) of Action of Metformin. Front. Endocrinol..

[47] Fontaine E. (2018). Metformin-Induced Mitochondrial Complex I Inhibition: Facts, Uncertainties, and Consequences. Front. Endocrinol..

[48] Christensen M.M., Hojlund K., Hother-Nielsen O., Stage T.B., Damkier P., Beck-Nielsen H., Brosen K. (2015). Steady-state pharmacokinetics of metformin is independent of the OCT1 genotype in healthy volunteers. Eur J. Clin. Pharm..

[49] Kinaan M., Ding H., Triggle C.R. (2015). Metformin: An Old Drug for the Treatment of Diabetes but a New Drug for the Protection of the Endothelium. Med. Princ. Pract..

[50] Liang X., Giacomini K.M. (2017). Transporters Involved in Metformin Pharmacokinetics and Treatment Response. J. Pharm. Sci..

[51] Choi M.K., Jin Q.R., Jin H.E., Shim C.K., Cho D.Y., Shin J.G., Song I.S. (2007). Effects of tetraalkylammonium compounds with different affinities for organic cation transporters on the pharmacokinetics of metformin. Biopharm. Drug Dispos..

[52] Kimura N., Masuda S., Tanihara Y., Ueo H., Okuda M., Katsura T., Inui K. (2005). Metformin is a superior substrate for renal organic cation transporter OCT2 rather than hepatic OCT1. Drug Metab. Pharmacokinet..

[53] Li S., Chen Y., Zhang S., More S.S., Huang X., Giacomini K.M. (2011). Role of organic cation transporter 1, OCT1 in the pharmacokinetics and toxicity of cis-diammine(pyridine)chloroplatinum(II) and oxaliplatin in mice. Pharm. Res..

[54] Chen E.C., Liang X., Yee S.W., Geier E.G., Stocker S.L., Chen L., Giacomini K.M. (2015). Targeted disruption of organic cation transporter 3 attenuates the pharmacologic response to metformin. Mol. Pharmacol..

[55] Chen Y., Teranishi K., Li S., Yee S.W., Hesselson S., Stryke D., Johns S.J., Ferrin T.E., Kwok P., Giacomini K.M. (2009). Genetic variants in multidrug and toxic compound extrusion-1, hMATE1, alter transport function. Pharm. J..

[56] Masuda S., Terada T., Yonezawa A., Tanihara Y., Kishimoto K., Katsura T., Ogawa O., Inui K. (2006). Identification and functional characterization of a new human kidney-specific H+/organic cation antiporter, kidney-specific multidrug and toxin extrusion 2. J. Am. Soc. Nephrol..

[57] Zhou M., Xia L., Wang J. (2007). Metformin transport by a newly cloned proton-stimulated organic cation transporter (plasma membrane monoamine transporter) expressed in human intestine. Drug Metab. Dispos..

[58] Liang X., Chien H.C., Yee S.W., Giacomini M.M., Chen E.C., Piao M., Hao J., Twelves J., Lepist E.I., Ray A.S. (2015). Metformin Is a Substrate and Inhibitor of the Human Thiamine Transporter, THTR-2 (SLC19A3). Mol. Pharm..

[59] Shu Y., Brown C., Castro R.A., Shi R.J., Lin E.T., Owen R.P., Sheardown S.A., Yue L., Burchard E.G., Brett C.M. (2008). Effect of genetic variation in the organic cation transporter 1, OCT1, on metformin pharmacokinetics. Clin. Pharm..

[60] Shu Y., Sheardown S.A., Brown C., Owen R.P., Zhang S., Castro R.A., Ianculescu A.G., Yue L., Lo J.C., Burchard E.G. (2007). Effect of genetic variation in the organic cation transporter 1 (OCT1) on metformin action. J. Clin. Investig..

[61] Cai H., Zhang Y., Han T.K., Everett R.S., Thakker D.R. (2016). Cation-selective transporters are critical to the AMPK-mediated antiproliferative effects of metformin in human breast cancer cells. Int J. Cancer.

[62] Checkley L.A., Rudolph M.C., Wellberg E.A., Giles E.D., Wahdan-Alaswad R.S., Houck J.A., Edgerton S.M., Thor A.D., Schedin P., Anderson S.M. (2017). Metformin Accumulation Correlates with Organic Cation Transporter 2 Protein Expression and Predicts Mammary Tumor Regression in Vivo. Cancer Prev. Res..

[63] Chowdhury S., Yung E., Pintilie M., Muaddi H., Chaib S., Yeung M., Fusciello M., Sykes J., Pitcher B., Hagenkort A. (2016). MATE2 Expression Is Associated with Cancer Cell Response to Metformin. PLoS ONE.

[64] Cai H., Everett R.S., Thakker D.R. (2019). Efficacious dose of metformin for breast cancer therapy is determined by cation transporter expression in tumours. Br. J. Pharmacol..

[65] Rahmani J., Manzari N., Thompson J., Gudi S.K., Chhabra M., Naik G., Mousavi S.M., Varkaneh H.K., Clark C., Zhang Y. (2019). The effect of metformin on biomarkers associated with breast cancer outcomes: A systematic review, meta-analysis, and dose-response of randomized clinical trials. Clin. Transl. Oncol..

[66] Le A., Lane A.N., Hamaker M., Bose S., Gouw A., Barbi J., Tsukamoto T., Rojas C.J., Slusher B.S., Zhang H. (2012). Glucose-Independent Glutamine Metabolism via TCA Cycling for Proliferation and Survival in B Cells. Cell Metab..

[67] Metallo C.M., Gameiro P.A., Bell E.L., Mattaini K.R., Yang J., Hiller K., Jewell C.M., Johnson Z.R., Irvine D.J., Guarente L. (2012). Reductive glutamine metabolism by IDH1 mediates lipogenesis under hypoxia. Nature.

[68] Samuel S.M., Ghosh S., Majeed Y., Arunachalam G., Emara M.M., Ding H., Triggle C.R. (2017). Metformin represses glucose starvation induced autophagic response in microvascular endothelial cells and promotes cell death. Biochem. Pharmacol..

[69] Karnevi E., Said K., Andersson R., Rosendahl A.H. (2013). Metformin-mediated growth inhibition involves suppression of the IGF-I receptor signalling pathway in human pancreatic cancer cells. BMC Cancer.

[70] Lee J., Hong E.M., Kim J.H., Jung J.H., Park S.W., Koh D.H., Choi M.H., Jang H.J., Kae S.H. (2019). Metformin Induces Apoptosis and Inhibits Proliferation through the AMP-Activated Protein Kinase and Insulin-like Growth Factor 1 Receptor Pathways in the Bile Duct Cancer Cells. J. Cancer.

[71] Pernicova I., Korbonits M. (2014). Metformin—mode of action and clinical implications for diabetes and cancer. Nat. Rev. Endocrinol..

[72] Rojas L.B., Gomes M.B. (2013). Metformin: An old but still the best treatment for type 2 diabetes. Diabetol. Metab. Syndr..

[73] Triggle C.R., Ding H. (2017). Metformin is not just an antihyperglycaemic drug but also has protective effects on the vascular endothelium. Acta Physiol..

[74] Ferroni P., Riondino S., Buonomo O., Palmirotta R., Guadagni F., Roselli M. (2015). Type 2 Diabetes and Breast Cancer: The Interplay between Impaired Glucose Metabolism and Oxidant Stress. Oxid. Med. Cell Longev..

[75] Paquette M., El-Houjeiri L., Pause A. (2018). mTOR Pathways in Cancer and Autophagy. Cancers.

[76] Tian T., Li X., Zhang J. (2019). mTOR Signaling in Cancer and mTOR Inhibitors in Solid Tumor Targeting Therapy. Int J. Mol. Sci..

[77] Conciatori F., Bazzichetto C., Falcone I., Pilotto S., Bria E., Cognetti F., Milella M., Ciuffreda L. (2018). Role of mTOR Signaling in Tumor Microenvironment: An Overview. Int J. Mol. Sci..

[78] Chen H., Liu H., Qing G. (2018). Targeting oncogenic Myc as a strategy for cancer treatment. Signal. Transduct. Target. Ther..

[79] Gabay M., Li Y., Felsher D.W. (2014). MYC activation is a hallmark of cancer initiation and maintenance. Cold Spring Harb. Perspect. Med..

[80] Elbadawy M., Usui T., Yamawaki H., Sasaki K. (2019). Emerging Roles of C-Myc in Cancer Stem Cell-Related Signaling and Resistance to Cancer Chemotherapy: A Potential Therapeutic Target Against Colorectal Cancer. Int J. Mol. Sci..

[81] Park M.H., Hong J.T. (2016). Roles of NF-κB in Cancer and Inflammatory Diseases and Their Therapeutic Approaches. Cells.

[82] Xia Y., Shen S., Verma I.M. (2014). NF-κB, an active player in human cancers. Cancer Immunol. Res..

[83] Li F., Zhang J., Arfuso F., Chinnathambi A., Zayed M.E., Alharbi S.A., Kumar A.P., Ahn K.S., Sethi G. (2015). NF-κB in cancer therapy. Arch. Toxicol..

[84] Ozaki T., Nakagawara A. (2011). Role of p53 in Cell Death and Human Cancers. Cancers.

[85] Blandino G., Di Agostino S. (2018). New therapeutic strategies to treat human cancers expressing mutant p53 proteins. J. Exp. Clin. Cancer Res..

[86] Zhou X., Hao Q., Lu H. (2018). Mutant p53 in cancer therapy—the barrier or the path. J. Mol. Cell Biol..

[87] Lai H.-H., Li J.-N., Wang M.-Y., Huang H.-Y., Croce C.M., Sun H.-L., Lyu Y.-J., Kang J.-W., Chiu C.-F., Hung M.-C. (2018). HIF-1α promotes autophagic proteolysis of Dicer and enhances tumor metastasis. J. Clin. Investig..

[88] Villanueva T. (2010). It’s nicer with DICER. Nat. Rev. Cancer.

[89] Martello G., Rosato A., Ferrari F., Manfrin A., Cordenonsi M., Dupont S., Enzo E., Guzzardo V., Rondina M., Spruce T. (2010). A MicroRNA Targeting Dicer for Metastasis Control. Cell.

[90] Hawley S.A., Gadalla A.E., Olsen G.S., Hardie D.G. (2002). The Antidiabetic Drug Metformin Activates the AMP-Activated Protein Kinase Cascade via an Adenine Nucleotide-Independent Mechanism. Diabetes.

[91] Zhou G., Myers R., Li Y., Chen Y., Shen X., Fenyk-Melody J., Wu M., Ventre J., Doebber T., Fujii N. (2001). Role of AMP-activated protein kinase in mechanism of metformin action. J. Clin. Investig..

[92] Meng S., Cao J., He Q., Xiong L., Chang E., Radovick S., Wondisford F.E., He L. (2015). Metformin activates AMP-activated protein kinase by promoting formation of the αβγ heterotrimeric complex. J. Biol. Chem..

[93] Howell J.J., Hellberg K., Turner M., Talbott G., Kolar M.J., Ross D.S., Hoxhaj G., Saghatelian A., Shaw R.J., Manning B.D. (2017). Metformin Inhibits Hepatic mTORC1 Signaling via Dose-Dependent Mechanisms Involving AMPK and the TSC Complex. Cell Metab..

[94] Sabnis H.S., Somasagara R.R., Bunting K.D. (2017). Targeting MYC Dependence by Metabolic Inhibitors in Cancer. Genes.

[95] Shen P., Reineke L.C., Knutsen E., Chen M., Pichler M., Ling H., Calin G.A. (2018). Metformin blocks MYC protein synthesis in colorectal cancer via mTOR-4EBP-eIF4E and MNK1-eIF4G-eIF4E signaling. Mol. Oncol..

[96] Hattori Y., Suzuki K., Hattori S., Kasai K. (2006). Metformin Inhibits Cytokine-Induced Nuclear Factor κB Activation Via AMP-Activated Protein Kinase Activation in Vascular Endothelial Cells. Hypertension.

[97] Sekino N., Kano M., Matsumoto Y., Sakata H., Akutsu Y., Hanari N., Murakami K., Toyozumi T., Takahashi M., Otsuka R. (2018). Antitumor effects of metformin are a result of inhibiting nuclear factor kappa B nuclear translocation in esophageal squamous cell carcinoma. Cancer Sci..

[98] Xu S., Yang Z., Jin P., Yang X., Li X., Wei X., Wang Y., Long S., Zhang T., Chen G. (2018). Metformin Suppresses Tumor Progression by Inactivating Stromal Fibroblasts in Ovarian Cancer. Mol. Cancer Ther..

[99] Li P., Zhao M., Parris A., Feng X., Yang X. (2015). P53 is required for metformin-induced growth inhibition, senescence and apoptosis in breast cancer cells. Biochem. Biophys. Res. Commun..

[100] Chen L., Ahmad N., Liu X. (2016). Combining p53 stabilizers with metformin induces synergistic apoptosis through regulation of energy metabolism in castration-resistant prostate cancer. Cell Cycle.

[101] Yi Y., Zhang W., Yi J., Xiao Z.-X. (2019). Role of p53 Family Proteins in Metformin Anti-Cancer Activities. J. Cancer.

[102] Yi G., He Z., Zhou X., Xian L., Yuan T., Jia X., Hong J., He L., Liu J. (2013). Low concentration of metformin induces a p53-dependent senescence in hepatoma cells via activation of the AMPK pathway. Int. J. Oncol..

[103] Noren Hooten N., Martin-Montalvo A., Dluzen D.F., Zhang Y., Bernier M., Zonderman A.B., Becker K.G., Gorospe M., de Cabo R., Evans M.K. (2016). Metformin-mediated increase in DICER1 regulates microRNA expression and cellular senescence. Aging Cell.

[104] Pulito C., Donzelli S., Muti P., Puzzo L., Strano S., Blandino G. (2014). microRNAs and cancer metabolism reprogramming: The paradigm of metformin. Ann. Transl. Med..

[105] Blandino G., Valerio M., Cioce M., Mori F., Casadei L., Pulito C., Sacconi A., Biagioni F., Cortese G., Galanti S. (2012). Metformin elicits anticancer effects through the sequential modulation of DICER and c-MYC. Nat. Commun..

[106] Gwinn D.M., Shackelford D.B., Egan D.F., Mihaylova M.M., Mery A., Vasquez D.S., Turk B.E., Shaw R.J. (2008). AMPK phosphorylation of raptor mediates a metabolic checkpoint. Mol. Cell.

[107] Dowling R.J.O., Zakikhani M., Fantus I.G., Pollak M., Sonenberg N. (2007). Metformin Inhibits Mammalian Target of Rapamycin–Dependent Translation Initiation in Breast Cancer Cells. Cancer Res..

[108] Sancak Y., Thoreen C.C., Peterson T.R., Lindquist R.A., Kang S.A., Spooner E., Carr S.A., Sabatini D.M. (2007). PRAS40 Is an Insulin-Regulated Inhibitor of the mTORC1 Protein Kinase. Mol. Cell.

[109] Shaw R.J. (2009). LKB1 and AMP-activated protein kinase control of mTOR signalling and growth. Acta Physiol..

[110] Inoki K., Zhu T., Guan K.-L. (2003). TSC2 Mediates Cellular Energy Response to Control Cell Growth and Survival. Cell.

[111] Fullerton M.D., Galic S., Marcinko K., Sikkema S., Pulinilkunnil T., Chen Z.-P., O’Neill H.M., Ford R.J., Palanivel R., O’Brien M. (2013). Single phosphorylation sites in Acc1 and Acc2 regulate lipid homeostasis and the insulin-sensitizing effects of metformin. Nat. Med..

[112] Gupta S., Roy A., Dwarakanath B.S. (2017). Metabolic Cooperation and Competition in the Tumor Microenvironment: Implications for Therapy. Front. Oncol..

[113] El-Mir M.Y., Nogueira V., Fontaine E., Averet N., Rigoulet M., Leverve X. (2000). Dimethylbiguanide inhibits cell respiration via an indirect effect targeted on the respiratory chain complex I. J. Biol. Chem..

[114] Owen M.R., Doran E., Halestrap A.P. (2000). Evidence that metformin exerts its anti-diabetic effects through inhibition of complex 1 of the mitochondrial respiratory chain. Biochem. J..

[115] Luengo A., Sullivan L.B., Heiden M.G. (2014). Understanding the complex-I-ty of metformin action: Limiting mitochondrial respiration to improve cancer therapy. BMC Biol..

[116] Koido M., Haga N., Furuno A., Tsukahara S., Sakurai J., Tani Y., Sato S., Tomida A. (2017). Mitochondrial deficiency impairs hypoxic induction of HIF-1 transcriptional activity and retards tumor growth. Oncotarget.

[117] Haga N., Saito S., Tsukumo Y., Sakurai J., Furuno A., Tsuruo T., Tomida A. (2010). Mitochondria regulate the unfolded protein response leading to cancer cell survival under glucose deprivation conditions. Cancer Sci..

[118] Ma L., Wei J., Wan J., Wang W., Wang L., Yuan Y., Yang Z., Liu X., Ming L. (2019). Low glucose and metformin-induced apoptosis of human ovarian cancer cells is connected to ASK1 via mitochondrial and endoplasmic reticulum stress-associated pathways. J. Exp. Clin. Cancer Res..

[119] Malhotra J.D., Kaufman R.J. (2011). ER stress and its functional link to mitochondria: Role in cell survival and death. Cold Spring Harb. Perspect Biol..

[120] Wolff N.C., Vega-Rubin-de-Celis S., Xie X.-J., Castrillon D.H., Kabbani W., Brugarolas J. (2011). Cell-Type-Dependent Regulation of mTORC1 by REDD1 and the Tumor Suppressors TSC1/TSC2 and LKB1 in Response to Hypoxia. Mol. Cell. Biol..

[121] Katiyar S., Liu E., Knutzen C.A., Lang E.S., Lombardo C.R., Sankar S., Toth J.I., Petroski M.D., Ronai Z.e., Chiang G.G. (2009). REDD1, an inhibitor of mTOR signalling, is regulated by the CUL4A-DDB1 ubiquitin ligase. EMBO Rep..

[122] Tirado-Hurtado I., Fajardo W., Pinto J.A. (2018). DNA Damage Inducible Transcript 4 Gene: The Switch of the Metabolism as Potential Target in Cancer. Front. Oncol..

[123] DeYoung M.P., Horak P., Sofer A., Sgroi D., Ellisen L.W. (2008). Hypoxia regulates TSC1/2-mTOR signaling and tumor suppression through REDD1-mediated 14-3-3 shuttling. Genes Dev..

[124] Ben Sahra I., Regazzetti C., Robert G., Laurent K., Le Marchand-Brustel Y., Auberger P., Tanti J.-F., Giorgetti-Peraldi S., Bost F. (2011). Metformin, Independent of AMPK, Induces mTOR Inhibition and Cell-Cycle Arrest through REDD1. Cancer Res..

[125] Lei Y., Yi Y., Liu Y., Liu X., Keller E.T., Qian C.-N., Zhang J., Lu Y. (2017). Metformin targets multiple signaling pathways in cancer. Chin. J. Cancer.

[126] Kim J., Kim E. (2016). Rag GTPase in amino acid signaling. Amino Acids.

[127] Nguyen T.P., Frank A.R., Jewell J.L. (2017). Amino acid and small GTPase regulation of mTORC1. Cell. Logist..

[128] Nicastro R., Sardu A., Panchaud N., De Virgilio C. (2017). The Architecture of the Rag GTPase Signaling Network. Biomolecules.

[129] Kalender A., Selvaraj A., Kim S.Y., Gulati P., Brûlé S., Viollet B., Kemp B.E., Bardeesy N., Dennis P., Schlager J.J. (2010). Metformin, independent of AMPK, inhibits mTORC1 in a rag GTPase-dependent manner. Cell Metab..

[130] Fu K., Jiang C., Huang X., Chan W.C., McKeithan T. (2013). The Combination Of 2-DG and Metformin Inhibits The mTORC1 Pathway and Suppresses Aggressive B Cell Lymphoma Growth and Survival. Blood.

[131] Avalle L., Camporeale A., Camperi A., Poli V. (2017). STAT3 in cancer: A double edged sword. Cytokine.

[132] Huynh J., Chand A., Gough D., Ernst M. (2019). Therapeutically exploiting STAT3 activity in cancer—using tissue repair as a road map. Nat. Rev. Cancer.

[133] Saengboonmee C., Seubwai W., Cha’on U., Sawanyawisuth K., Wongkham S., Wongkham C. (2017). Metformin Exerts Antiproliferative and Anti-metastatic Effects Against Cholangiocarcinoma Cells by Targeting STAT3 and NF-kB. Anticancer Res..

[134] Deng X.S., Wang S., Deng A., Liu B., Edgerton S.M., Lind S.E., Wahdan-Alaswad R., Thor A.D. (2012). Metformin targets Stat3 to inhibit cell growth and induce apoptosis in triple-negative breast cancers. Cell Cycle.

[135] Varghese E., Samuel M.S., Abotaleb M., Cheema S., Mamtani R., Büsselberg D. (2018). The “Yin and Yang” of Natural Compounds in Anticancer Therapy of Triple-Negative Breast Cancers. Cancers.

[136] Dowling R.J.O., Niraula S., Chang M.C., Done S.J., Ennis M., McCready D.R., Leong W.L., Escallon J.M., Reedijk M., Goodwin P.J. (2015). Changes in insulin receptor signaling underlie neoadjuvant metformin administration in breast cancer: A prospective window of opportunity neoadjuvant study. Breast Cancer Res..

[137] De A., Kuppusamy G. (2019). Metformin in breast cancer: Preclinical and clinical evidence. Curr. Probl. Cancer.

[138] Menendez J.A., Oliveras-Ferraros C., Cufi S., Corominas-Faja B., Joven J., Martin-Castillo B., Vazquez-Martin A. (2012). Metformin is synthetically lethal with glucose withdrawal in cancer cells. Cell Cycle.

[139] Wahdan-Alaswad R., Fan Z., Edgerton S.M., Liu B., Deng X.S., Arnadottir S.S., Richer J.K., Anderson S.M., Thor A.D. (2013). Glucose promotes breast cancer aggression and reduces metformin efficacy. Cell Cycle.

[140] Silvestri A., Palumbo F., Rasi I., Posca D., Pavlidou T., Paoluzi S., Castagnoli L., Cesareni G. (2015). Metformin Induces Apoptosis and Downregulates Pyruvate Kinase M2 in Breast Cancer Cells Only When Grown in Nutrient-Poor Conditions. PLoS ONE.

[141] Zordoky B.N., Bark D., Soltys C.L., Sung M.M., Dyck J.R. (2014). The anti-proliferative effect of metformin in triple-negative MDA-MB-231 breast cancer cells is highly dependent on glucose concentration: Implications for cancer therapy and prevention. Biochim. Biophys. Acta.

[142] Queiroz E.A., Puukila S., Eichler R., Sampaio S.C., Forsyth H.L., Lees S.J., Barbosa A.M., Dekker R.F., Fortes Z.B., Khaper N. (2014). Metformin induces apoptosis and cell cycle arrest mediated by oxidative stress, AMPK and FOXO3a in MCF-7 breast cancer cells. PLoS ONE.

[143] Ben Sahra I., Laurent K., Loubat A., Giorgetti-Peraldi S., Colosetti P., Auberger P., Tanti J.F., Le Marchand-Brustel Y., Bost F. (2008). The antidiabetic drug metformin exerts an antitumoral effect in vitro and in vivo through a decrease of cyclin D1 level. Oncogene.

[144] Liu B., Fan Z., Edgerton S.M., Deng X.S., Alimova I.N., Lind S.E., Thor A.D. (2009). Metformin induces unique biological and molecular responses in triple negative breast cancer cells. Cell Cycle.

[145] Wahdan-Alaswad R.S., Edgerton S.M., Salem H.S., Thor A.D. (2018). Metformin Targets Glucose Metabolism in Triple Negative Breast Cancer. J. Oncol. Transl. Res..

[146] Wang J.-C., Li G.-Y., Wang B., Han S.-X., Sun X., Jiang Y.-N., Shen Y.-W., Zhou C., Feng J., Lu S.-Y. (2019). Metformin inhibits metastatic breast cancer progression and improves chemosensitivity by inducing vessel normalization via PDGF-B downregulation. J. Exp. Clin. Cancer Res..

[147] Andrzejewski S., Gravel S.-P., Pollak M., St-Pierre J. (2014). Metformin directly acts on mitochondria to alter cellular bioenergetics. Cancer Metab..

[148] Xu H., Aldrich M.C., Chen Q., Liu H., Peterson N.B., Dai Q., Levy M., Shah A., Han X., Ruan X. (2015). Validating drug repurposing signals using electronic health records: A case study of metformin associated with reduced cancer mortality. J. Am. Med. Inf. Assoc..

[149] Hatoum D., McGowan E.M. (2015). Recent Advances in the Use of Metformin: Can Treating Diabetes Prevent Breast Cancer?. Biomed. Res. Int..

[150] Chlebowski R.T., McTiernan A., Wactawski-Wende J., Manson J.E., Aragaki A.K., Rohan T., Ipp E., Kaklamani V.G., Vitolins M., Wallace R. (2012). Diabetes, metformin, and breast cancer in postmenopausal women. J. Clin. Oncol..

[151] Gonzalez-Angulo A.M., Meric-Bernstam F. (2010). Metformin: A therapeutic opportunity in breast cancer. Clin. Cancer Res..

[152] Campagnoli C., Berrino F., Venturelli E., Abba C., Biglia N., Brucato T., Cogliati P., Danese S., Donadio M., Zito G. (2013). Metformin decreases circulating androgen and estrogen levels in nondiabetic women with breast cancer. Clin. Breast Cancer.

[153] Campagnoli C., Pasanisi P., Abba C., Ambroggio S., Biglia N., Brucato T., Colombero R., Danese S., Donadio M., Venturelli E. (2012). Effect of different doses of metformin on serum testosterone and insulin in non-diabetic women with breast cancer: A randomized study. Clin. Breast Cancer.

[154] Jiralerspong S., Palla S.L., Giordano S.H., Meric-Bernstam F., Liedtke C., Barnett C.M., Hsu L., Hung M.C., Hortobagyi G.N., Gonzalez-Angulo A.M. (2009). Metformin and pathologic complete responses to neoadjuvant chemotherapy in diabetic patients with breast cancer. J. Clin. Oncol..

[155] Sonnenblick A., Agbor-Tarh D., Bradbury I., Di Cosimo S., Azim H.A., Fumagalli D., Sarp S., Wolff A.C., Andersson M., Kroep J. (2017). Impact of Diabetes, Insulin, and Metformin Use on the Outcome of Patients With Human Epidermal Growth Factor Receptor 2-Positive Primary Breast Cancer: Analysis From the ALTTO Phase III Randomized Trial. J. Clin. Oncol..

[156] Tang G.H., Satkunam M., Pond G.R., Steinberg G.R., Blandino G., Schunemann H.J., Muti P. (2018). Association of Metformin with Breast Cancer Incidence and Mortality in Patients with Type II Diabetes: A GRADE-Assessed Systematic Review and Meta-analysis. Cancer Epidemiol. Biomark. Prev..

[157] Esteva F.J., Moulder S.L., Gonzalez-Angulo A.M., Ensor J., Murray J.L., Green M.C., Koenig K.B., Lee M.H., Hortobagyi G.N., Yeung S.C. (2013). Phase I trial of exemestane in combination with metformin and rosiglitazone in nondiabetic obese postmenopausal women with hormone receptor-positive metastatic breast cancer. Cancer Chemother Pharm..

[158] Kalinsky K., Crew K.D., Refice S., Xiao T., Wang A., Feldman S.M., Taback B., Ahmad A., Cremers S., Hibshoosh H. (2014). Presurgical trial of metformin in overweight and obese patients with newly diagnosed breast cancer. Cancer Investig..

[159] Kalinsky K., Zheng T., Hibshoosh H., Du X., Mundi P., Yang J., Refice S., Feldman S.M., Taback B., Connolly E. (2017). Proteomic modulation in breast tumors after metformin exposure: Results from a “window of opportunity” trial. Clin. Transl. Oncol..

[160] Lord S.R., Cheng W.C., Liu D., Gaude E., Haider S., Metcalf T., Patel N., Teoh E.J., Gleeson F., Bradley K. (2018). Integrated Pharmacodynamic Analysis Identifies Two Metabolic Adaption Pathways to Metformin in Breast Cancer. Cell Metab..

[161] Kim J., Lim W., Kim E.K., Kim M.K., Paik N.S., Jeong S.S., Yoon J.H., Park C.H., Ahn S.H., Kim L.S. (2014). Phase II randomized trial of neoadjuvant metformin plus letrozole versus placebo plus letrozole for estrogen receptor positive postmenopausal breast cancer (METEOR). BMC Cancer.

[162] Lega I.C., Fung K., Austin P.C., Lipscombe L.L. (2017). Metformin and breast cancer stage at diagnosis: A population-based study. Curr. Oncol..

[163] Lega I.C., Austin P.C., Gruneir A., Goodwin P.J., Rochon P.A., Lipscombe L.L. (2013). Association Between Metformin Therapy and Mortality After Breast Cancer. Diabetes Care.

[164] Peng M., Darko K.O., Tao T., Huang Y., Su Q., He C., Yin T., Liu Z., Yang X. (2017). Combination of metformin with chemotherapeutic drugs via different molecular mechanisms. Cancer Treat. Rev..

[165] Bozic I., Reiter J.G., Allen B., Antal T., Chatterjee K., Shah P., Moon Y.S., Yaqubie A., Kelly N., Le D.T. (2013). Evolutionary dynamics of cancer in response to targeted combination therapy. eLife.

[166] Chatterjee S., Thaker N., De A. (2015). Combined 2-deoxy glucose and metformin improves therapeutic efficacy of sodium-iodide symporter-mediated targeted radioiodine therapy in breast cancer cells. Breast Cancer.

[167] Arbe M.F., Fondello C., Agnetti L., Álvarez G.M., Tellado M.N., Glikin G.C., Finocchiaro L.M.E., Villaverde M.S. (2017). Inhibition of bioenergetic metabolism by the combination of metformin and 2-deoxyglucose highly decreases viability of feline mammary carcinoma cells. Res. Vet. Sci..

[168] Bizjak M., Malavašič P., Dolinar K., Pohar J., Pirkmajer S., Pavlin M. (2017). Combined treatment with Metformin and 2-deoxy glucose induces detachment of viable MDA-MB-231 breast cancer cells in vitro. Sci. Rep..

[169] Wokoun U., Hellriegel M., Emons G., Grundker C. (2017). Co-treatment of breast cancer cells with pharmacologic doses of 2-deoxy-D-glucose and metformin: Starving tumors. Oncol. Rep..

[170] Xue C., Wang C., Sun Y., Meng Q., Liu Z., Huo X., Sun P., Sun H., Ma X., Ma X. (2017). Targeting P-glycoprotein function, p53 and energy metabolism: Combination of metformin and 2-deoxyglucose reverses the multidrug resistance of MCF-7/Dox cells to doxorubicin. Oncotarget.

[171] Soo J.S.-S., Ng C.-H., Tan S.H., Malik R.A., Teh Y.-C., Tan B.-S., Ho G.-F., See M.-H., Taib N.A.M., Yip C.-H. (2015). Metformin synergizes 5-fluorouracil, epirubicin, and cyclophosphamide (FEC) combination therapy through impairing intracellular ATP production and DNA repair in breast cancer stem cells. Apoptosis.

[172] Zhao M., Wang Y., Du C., Liu Y., Zhang N., Luo F. (2018). Aspirin and metformin exhibit antitumor activity in murine breast cancer. Oncol. Rep..

[173] Amaral M.E.A., Nery L.R., Leite C.E., de Azevedo Junior W.F., Campos M.M. (2018). Pre-clinical effects of metformin and aspirin on the cell lines of different breast cancer subtypes. Investig. New Drugs.

[174] Rasouli S., Zarghami N. (2018). Synergistic Growth Inhibitory Effects of Chrysin and Metformin Combination on Breast Cancer Cells through hTERT and Cyclin D1 Suppression. Asian Pac. J. Cancer Prev..

[175] Falah R.R., Talib W.H., Shbailat S.J. (2017). Combination of metformin and curcumin targets breast cancer in mice by angiogenesis inhibition, immune system modulation and induction of p53 independent apoptosis. Ther. Adv. Med. Oncol..

[176] Farajzadeh R., Pilehvar-Soltanahmadi Y., Dadashpour M., Javidfar S., Lotfi-Attari J., Sadeghzadeh H., Shafiei-Irannejad V., Zarghami N. (2018). Nano-encapsulated metformin-curcumin in PLGA/PEG inhibits synergistically growth and hTERT gene expression in human breast cancer cells. Artif. Cells Nanomed. Biotechnol..

[177] Cuyàs E., Martin-Castillo B., Bosch-Barrera J., Menendez J.A. (2017). Metformin inhibits RANKL and sensitizes cancer stem cells to denosumab. Cell Cycle.

[178] Haugrud A.B., Zhuang Y., Coppock J.D., Miskimins W.K. (2014). Dichloroacetate enhances apoptotic cell death via oxidative damage and attenuates lactate production in metformin-treated breast cancer cells. Breast Cancer Res. Treat..

[179] Hong S.-E., Jin H.-O., Kim H.-A., Seong M.-K., Kim E.-K., Ye S.-K., Choe T.-B., Lee J.K., Kim J.-I., Park I.-C. (2016). Targeting HIF-1α is a prerequisite for cell sensitivity to dichloroacetate (DCA) and metformin. Biochem. Biophys. Res. Commun..

[180] Cooper A.C., Fleming I.N., Phyu S.M., Smith T.A.D. (2015). Changes in [18F]Fluoro-2-deoxy-d-glucose incorporation induced by doxorubicin and anti-HER antibodies by breast cancer cells modulated by co-treatment with metformin and its effects on intracellular signalling. J. Cancer Res. Clin. Oncol..

[181] Lu Z., Long Y., Cun X., Wang X., Li J., Mei L., Yang Y., Li M., Zhang Z., He Q. (2018). A size-shrinkable nanoparticle-based combined anti-tumor and anti-inflammatory strategy for enhanced cancer therapy. Nanoscale.

[182] Shafiei-Irannejad V., Samadi N., Salehi R., Yousefi B., Rahimi M., Akbarzadeh A., Zarghami N. (2018). Reversion of Multidrug Resistance by Co-Encapsulation of Doxorubicin and Metformin in Poly(lactide-co-glycolide)-d-α-tocopheryl Polyethylene Glycol 1000 Succinate Nanoparticles. Pharm. Res..

[183] Shafiei-Irannejad V., Samadi N., Yousefi B., Salehi R., Velaei K., Zarghami N. (2018). Metformin enhances doxorubicin sensitivity via inhibition of doxorubicin efflux in P-gp-overexpressing MCF-7 cells. Chem. Biol. Drug Des..

[184] El-Ashmawy N.E., Khedr N.F., El-Bahrawy H.A., Abo Mansour H.E. (2017). Metformin augments doxorubicin cytotoxicity in mammary carcinoma through activation of adenosine monophosphate protein kinase pathway. Tumor Biol..

[185] Li Y., Wang M., Zhi P., You J., Gao J.-Q. (2018). Metformin synergistically suppress tumor growth with doxorubicin and reverse drug resistance by inhibiting the expression and function of P-glycoprotein in MCF7/ADR cells and xenograft models. Oncotarget.

[186] Lau Y.-K.I., Du X., Rayannavar V., Hopkins B., Shaw J., Bessler E., Thomas T., Pires M.M., Keniry M., Parsons R.E. (2014). Metformin and erlotinib synergize to inhibit basal breast cancer. Oncotarget.

[187] Ariaans G., Jalving M., Vries E.G.E.d., Jong S.d. (2017). Anti-tumor effects of everolimus and metformin are complementary and glucose-dependent in breast cancer cells. BMC Cancer.

[188] Wang Y., Wei J., Li L., Fan C., Sun Y. (2014). Combined Use of Metformin and Everolimus Is Synergistic in the Treatment of Breast Cancer Cells. Oncol. Res..

[189] Zheng Z., Zhu W., Yang B., Chai R., Liu T., Li F., Ren G., Ji S., Liu S., Li G. (2018). The co-treatment of metformin with flavone synergistically induces apoptosis through inhibition of PI3K/AKT pathway in breast cancer cells. Oncol. Lett..

[190] Bojková B., Kajo K., Kisková T., Kubatka P., Žúbor P., Solár P., Péč M., Adamkov M. (2018). Metformin and melatonin inhibit DMBA-induced mammary tumorigenesis in rats fed a high-fat diet. Anti-Cancer Drugs.

[191] Xiao Y., Wang S., Zong Q., Yin Z. (2018). Co-delivery of Metformin and Paclitaxel Via Folate-Modified pH-Sensitive Micelles for Enhanced Anti-tumor Efficacy. AAPS PharmSciTech.

[192] Chatran M., Pilehvar-Soltanahmadi Y., Dadashpour M., Faramarzi L., Rasouli S., Jafari-Gharabaghlou D., Asbaghi N., Zarghami N. (2018). Synergistic anti-proliferative effects of metformin and silibinin combination on T47D breast cancer cells via hTERT and cyclin D1 inhibition. Drug Res..

[193] Yeo S.K., Paul R., Haas M., Wang C., Guan J.-L. (2018). Improved efficacy of mitochondrial disrupting agents upon inhibition of autophagy in a mouse model of BRCA1-deficient breast cancer. Autophagy.

[194] Ma J., Guo Y., Chen S., Zhong C., Xue Y., Zhang Y., Lai X., Wei Y., Yu S., Zhang J. (2014). Metformin enhances tamoxifen-mediated tumor growth inhibition in ER-positive breast carcinoma. BMC Cancer.

[195] Banala V.T., Sharma S., Barnwal P., Urandur S., Shukla R.P., Ahmad N., Mittapelly N., Pandey G., Dwivedi M., Kalleti N. (2018). Synchronized Ratiometric Codelivery of Metformin and Topotecan through Engineered Nanocarrier Facilitates In Vivo Synergistic Precision Levels at Tumor Site. Adv. Healthc. Mater..

[196] Guo L.S., Li H.X., Li C.Y., Zhang S.Y., Chen J., Wang Q.L., Gao J.M., Liang J.Q., Gao M.T., Wu Y.J. (2015). Synergistic antitumor activity of vitamin D3 combined with metformin in human breast carcinoma MDA-MB-231 cells involves m-TOR related signaling pathways. Pharmazie.

[197] Rao M., Gao C., Guo M., Law B.Y.K., Xu Y. (2018). Effects of metformin treatment on radiotherapy efficacy in patients with cancer and diabetes: A systematic review and meta-analysis. Cancer Manag. Res..

[198] Ferro A., Goyal S., Kim S., Wu H., Taunk N.K., Schiff D., Pirlamarla A., Haffty B.G. (2013). Evaluation of Diabetic Patients with Breast Cancer Treated with Metformin during Adjuvant Radiotherapy. Int. J. Breast Cancer.

[199] Keywan M., Dheyauldeen S., Ahmed E.M., Masoud N., Bagher F. (2019). Metformin as a Radiation Modifier; Implications to Normal Tissue Protection and Tumor Sensitization. Curr. Clin. Pharmacol..

[200] Brown S.L., Kolozsvary A., Isrow D.M., Al Feghali K., Lapanowski K., Jenrow K.A., Kim J.H. (2019). A Novel Mechanism of High Dose Radiation Sensitization by Metformin. Front. Oncol..

[201] Suissa S., Azoulay L. (2014). Metformin and Cancer: Mounting Evidence Against an Association. Diabetes Care.

[202] Mamtani R., Pfanzelter N., Haynes K., Finkelman B.S., Wang X., Keefe S.M., Haas N.B., Vaughn D.J., Lewis J.D. (2014). Incidence of Bladder Cancer in Patients With Type 2 Diabetes Treated With Metformin or Sulfonylureas. Diabetes Care.

[203] Samuel S.M., Satheesh N.J., Ghosh S., Büsselberg D., Majeed Y., Ding H., Triggle C.R. (2019). Treatment with a Combination of Metformin and 2-Deoxyglucose Upregulates Thrombospondin-1 in Microvascular Endothelial Cells: Implications in Anti-Angiogenic Cancer Therapy. Cancers.

[204] Kasznicki J., Sliwinska A., Drzewoski J. (2014). Metformin in cancer prevention and therapy. Ann. Transl. Med..

[205] Franciosi M., Lucisano G., Lapice E., Strippoli G.F.M., Pellegrini F., Nicolucci A. (2013). Metformin therapy and risk of cancer in patients with type 2 diabetes: Systematic review. PLoS ONE.

[206] Suissa S., Azoulay L. (2012). Metformin and the Risk of Cancer. Diabetes Care.

[207] Martin-Castillo B., Vazquez-Martin A., Oliveras-Ferraros C., Menendez J.A. (2010). Metformin and cancer: Doses, mechanisms and the dandelion and hormetic phenomena. Cell Cycle.

[208] Aljofan M., Riethmacher D. (2019). Anticancer activity of metformin: A systematic review of the literature. Future Sci. OA.

[209] Scherbakov A.M., Sorokin D.V., Tatarskiy V.V., Prokhorov N.S., Semina S.E., Berstein L.M., Krasil’nikov M.A. (2016). The phenomenon of acquired resistance to metformin in breast cancer cells: The interaction of growth pathways and estrogen receptor signaling. IUBMB Life.

[210] National Cancer Institute. https://www.cancer.gov/about-cancer/diagnosis-staging/diagnosis/tumor-markers-fact-sheet.

[211] National Cancer Institute. https://www.cancer.gov/about-cancer/causes-prevention/genetics/brca-fact-sheet.

[212] Li Y., Li J., Wang Y., Zhang Y., Chu J., Sun C., Fu Z., Huang Y., Zhang H., Yuan H. (2017). Roles of cancer/testis antigens (CTAs) in breast cancer. Cancer Lett..

[213] Fratta E., Coral S., Covre A., Parisi G., Colizzi F., Danielli R., Nicolay H.J., Sigalotti L., Maio M. (2011). The biology of cancer testis antigens: Putative function, regulation and therapeutic potential. Mol. Oncol..

[214] Gjerstorff M.F., Andersen M.H., Ditzel H.J. (2015). Oncogenic cancer/testis antigens: Prime candidates for immunotherapy. Oncotarget.

[215] Ademuyiwa F.O., Bshara W., Attwood K., Morrison C., Edge S.B., Karpf A.R., James S.A., Ambrosone C.B., O’Connor T.L., Levine E.G. (2012). NY-ESO-1 cancer testis antigen demonstrates high immunogenicity in triple negative breast cancer. PLoS ONE.

[216] Badovinac Crnjevic T., Spagnoli G., Juretic A., Jakic-Razumovic J., Podolski P., Saric N. (2012). High expression of MAGE-A10 cancer-testis antigen in triple-negative breast cancer. Med. Oncol..

[217] Balafoutas D., zur Hausen A., Mayer S., Hirschfeld M., Jaeger M., Denschlag D., Gitsch G., Jungbluth A., Stickeler E. (2013). Cancer testis antigens and NY-BR-1 expression in primary breast cancer: Prognostic and therapeutic implications. BMC Cancer.

[218] Curigliano G., Viale G., Ghioni M., Jungbluth A.A., Bagnardi V., Spagnoli G.C., Neville A.M., Nole F., Rotmensz N., Goldhirsch A. (2011). Cancer-testis antigen expression in triple-negative breast cancer. Ann. Oncol..

[219] Mirandola L., Pedretti E., Figueroa J.A., Chiaramonte R., Colombo M., Chapman C., Grizzi F., Patrinicola F., Kast W.M., Nguyen D.D. (2017). Cancer testis antigen Sperm Protein 17 as a new target for triple negative breast cancer immunotherapy. Oncotarget.

[220] Rastgoosalami M., Memar B., Aledavood S.A., Fanipakdel A. (2016). Evaluation of MAGE-1 Cancer-Testis Antigen Expression in Invasive Breast Cancer and its Correlation with Prognostic Factors. Iran. J. Cancer Prev..

[221] Saini S., Jagadish N., Gupta A., Bhatnagar A., Suri A. (2013). A novel cancer testis antigen, A-kinase anchor protein 4 (AKAP4) is a potential biomarker for breast cancer. PLoS ONE.

[222] Taylor M., Bolton L.M., Johnson P., Elliott T., Murray N. (2007). Breast cancer is a promising target for vaccination using cancer-testis antigens known to elicit immune responses. Breast Cancer Res..

[223] Soffer D., Shi J., Chung J., Schottinger J.E., Wallner L.P., Chlebowski R.T., Lentz S.E., Haque R. (2015). Metformin and breast and gynecological cancer risk among women with diabetes. BMJ Open Diabetes Res. Care.

[224] Camacho L., Dasgupta A., Jiralerspong S. (2015). Metformin in breast cancer - an evolving mystery. Breast Cancer Res..

[225] Podo F., Buydens L.M.C., Degani H., Hilhorst R., Klipp E., Gribbestad I.S., Van Huffel S., van Laarhoven H.W., Luts J., Monleon D. (2010). Triple-negative breast cancer: Present challenges and new perspectives. Mol. Oncol..

